# GC-MS Phytochemical Profiling, Pharmacological Properties, and In Silico Studies of *Chukrasia velutina* Leaves: A Novel Source for Bioactive Agents

**DOI:** 10.3390/molecules25153536

**Published:** 2020-08-02

**Authors:** Israt Jahan, Marzia Rahman Tona, Sanjida Sharmin, Mohammed Aktar Sayeed, Fatamatuz Zuhura Tania, Arkajyoti Paul, Md. Nazim Uddin Chy, Ahmed Rakib, Talha Bin Emran, Jesus Simal-Gandara

**Affiliations:** 1 Department of Pharmacy, Faculty of Science and Engineering, International Islamic University Chittagong, Kumira, Chittagong 4318, Bangladesh; istiisrat@gmail.com (I.J.); marziaeva96@gmail.com (M.R.T.); sayeed_ustc@yahoo.com (M.A.S.); fatamaztaniaiiuc@gmail.com (F.Z.T.); nazim107282@gmail.com (M.N.U.C.); 2 Drug Discovery, GUSTO A Research Group, Chittagong 4203, Bangladesh; arka.bgctub@gmail.com; 3 Department of Pharmacy, BGC Trust University Bangladesh, Chittagong 4381, Bangladesh; 4 Department of Pharmacy, Faculty of Biological Sciences, University of Chittagong, Chittagong 4331, Bangladesh; rakib.pharmacy.cu@gmail.com; 5 Nutrition and Bromatology Group, Department of Analytical and Food Chemistry, Faculty of 21 Food Science and Technology, University of Vigo–Ourense Campus, E32004 Ourense, Spain

**Keywords:** *Chukrasia velutina*, neuropsychiatric effects, antidepressant, anxiolytic, sedative, ADME/T, GC-MS, molecular docking

## Abstract

*Chukrasia velutina* is a local medicinal plant commonly known as chikrassy in Bangladesh, India, China, and other South Asian countries. The leaves, bark, and seeds are vastly used as herbal medicine for fever and diarrhea, and its leaves essential oils are used for antimicrobial purposes. In this study, we discuss the neuropsychiatric properties of *C. velutina* leaves through several animal models, quantitative and qualitative phytochemical analysis, and computational approaches. Neuropsychiatric effects were performed in rodents on the methanolic extract of *C. velutina* leaves (MECVL). Antidepressant, anxiolytic, and sedative effects experimented through these rodent models were used such as the force swimming test (FST), tail suspension test (TST), hole board test (HBT), elevated plus maze test (EPMT), light/dark box test (LDBT), open field test (OFT), and hole cross test (HCT). In these rodent models, 200 and 400 mg/kg doses were used which exhibited a significant result in the force swimming and tail suspension test (*p* < 0.001) for the antidepressant effect. In the anxiolytic study, the results were significant in the hole board, elevated plus maze, and light/dark box test (*p* < 0.001) for doses of 200 and 400 mg/kg. The result was also significant in the open field and hole cross test (*p* < 0.001) for sedative action in the sake of similar doses. Moreover, qualitative and quantitative studies were also performed through phytochemical screening and GC-MS analysis, and fifty-seven phytochemical compounds were found. These compounds were analyzed for pharmacokinetics properties using the SwissADME tool and from them, thirty-five compounds were considered for the molecular docking analysis. These phytoconstituents were docking against the human serotonin receptor, potassium channel receptor, and crystal structure of human beta-receptor, where eight of the compounds showed a good binding affinity towards the respective receptors considered to the reference standard drugs. After all of these analyses, it can be said that the secondary metabolite of *C. velutina* leaves (MECVL) could be a good source for inhibiting the neuropsychiatric disorders which were found on animal models as well as in computational studies.

## 1. Introduction

In recent years, neuropsychiatric disorders such as depression, anxiety, and insomnia play a great role in human behavior and mood. These disorders affect more than 9% of people around the world and their daily activities [1]. It is highlighted that neuropsychiatric disarrays are the third leading cause of inability according to the Global Burden of Disease, Injuries, and risk factors (GBD) when the case of suffering rates are exalted in women [2]. 

Depression is the most comprehensive illness among all disorders. Most people are suffering a lot due to depression and it was predicted that the rate of depression cases immensely increased in the upcoming few years [3]. Depression is the route factor of lots of diseases such as cardiac arrest, renal failure, stroke, diabetes, cancer, etc. It emits human willpower of living life and leads them to commit suicide [4]. In a few times, depression is a very early stage morbidity of Parkinson’s Disease (PD) [5]. Stress is a common cause of many CNS diseases. It creates depression and anxiety in the body by producing a free radical that turns the body into oxidative stress [3]. Moreover, anxiety is also a usual psychiatric problem caused by oxidative stress and affects a large number of people in this world [6]. When anxiety is in the severe stage, it is a huge CNS problem and a massive number of chronic diseases arise from it [7]. Performance impairment of numerous tasks are caused by anxiety disarrays and high rates of medically untold etiology are connected with enhancing the utilization of healthcare, that are sharply and independently attached with severe medical distemper, low levels of lifestyle, and inabilities [6]. Furthermore, uncontrolled and exorbitant worry has a considerable number of factors that varies from person to person and these troubles are experienced as getting easily fatigued, muscle tension, and sleep disturbance [8]. On the other hand, sleep disturbance causes insomnia, and that is the reason anxiety turns a human to an insomniac. Insomnia is beyond very numerous mental health problems in the current world that comprehends trouble in falling asleep, static sleep or waking too shortly, turning in the morning break that hampers normal people between 9% and 15% [9]. In addition, insomnia is also the result of post-traumatic stress disorder and obsessiveness. Chronic insomnia turns people restless and disables their thinking ability [10].

Currently, so many neuropsychiatric drugs should be used in anxiety, depression, insomnia, and other diseases related to psychiatric diseases. In those cases, these drugs cause side effects rather than efficacy [2,5,11]. For this purpose, researchers find the most effective drugs with minimum side effects [3,10]. In addition, Ayurveda plays a great role in phytotherapeutic regimens because it contains a large number of herbal plants that have a great source of phytochemical compounds and several numbers of compounds play a vital role for treating those neuropsychiatric disorders [12,13].

*Chukrasia velutina* A. Juss belongs in the Meliaceae family which is commonly known as chikrassy, Chittagong wood, lal devdari, Indian red wood, etc. [14] and the plant is widely spread in the forests of so many tropical countries such as Bangladesh, India, Myanmar, China, Laos, Thailand, Malaysia, and Vietnam [15]. This plant has different parts such as bark, leaves, stem, root bark, seeds, fruits, and all these parts have been traditionally used in the treatment of so many acute or chronic diseases such as fever and diarrhea, and the essential oil of leaves are used as antimalarial, antibacterial, and antifungal properties [16]. In the Hainan province in China, its root bark has been used as a traditional medicine for removing wind and heat from the body in a long time [17]. In the initial qualitative phytochemical analysis, *C. velutina* represents the arrival of certain phytochemical compounds of its leaves including resin, phenol, tannin, alkaloid, carbohydrate, and glycosides [7]. Furthermore, diverse pharmacological actions of this plant (leaves, root bark) have been reported previously. Kaur R. et al. narrated the inhibition of lipid peroxidation by extract and subfraction from its leaves and bark [14]. Nagalakshmi et al. reported the antimicrobial efficacy of leaves of the essential oil [16]. Wang et al. represented some chemical compounds tested for acetylcholinesterase and α-glucosidase that inhibited the capacity from the root bark of this plant [17].

The plant (*C. velutina*) has various useful medicinal properties, yet until now, no scientific investigation has been carried out to find out its effects against neuropsychiatric disorders such as anxiety, depression, and insomnia. Therefore, the current study has been designed to investigate the anxiolytic, antidepressant, and sedative effects of the methanolic extract of *C. velutina* leaves (MECVL) in various animal models. Then, a GC-MS (gas chromatography-mass spectrometry) analysis was also performed to identify the phytoconstituents present in the MECVL. Additionally, a computational study viz. in silico molecular docking, ADME/T properties was carried out to elucidate the molecular mechanism of the observed biological activities, and also to know its bioavailability and safety from the draggable point of view for the very first time.

## 2. Results

### 2.1. Qualitative Phytochemical Screening

Initiatory phytochemical screening of MECVL was a qualitative test which revealed the availability of resin, phenol, tannin, alkaloid, carbohydrate, and glycosides, and the result was mentioned in Table S1.

### 2.2. Gas Chromatography-Mass Spectroscopy (GC-MS) Analysis

**Figure 1:**
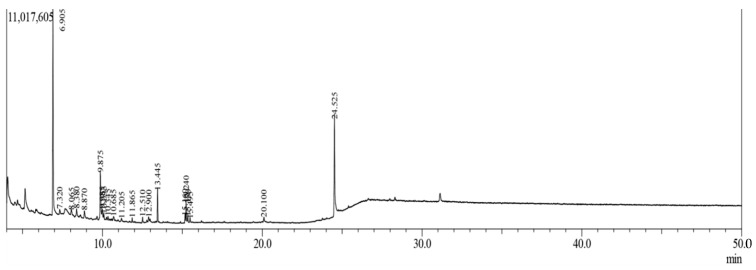
Total ionic chromatogram (TIC) of methanolic extract of *C. velutina* leaves (MECVL) by GC-MS.

The GC-MS analysis of MECVL transpired 57 compounds having retention times of between 6.902 and 24.520 min which were recorded in (Table 1) when the chromatogram was in Figure 1.

### 2.3. Acute Toxicity Test

All extended doses from 5 to 2000 mg/kg did not express the testimony of toxicity or behavioral oddities during the acute toxicity test. In addition, no fatality or physical abnormalities such as loss of body weight or allergic reaction, etc. were executed for three days after oral administration of MECVL which reported that the acute toxic profile up to 2000 mg/kg was not present in this extract (data not shown).

### 2.4. Antidepressant Activity

#### 2.4.1. Effect of MECVL on Force Swimming Test

**Figure 2:**
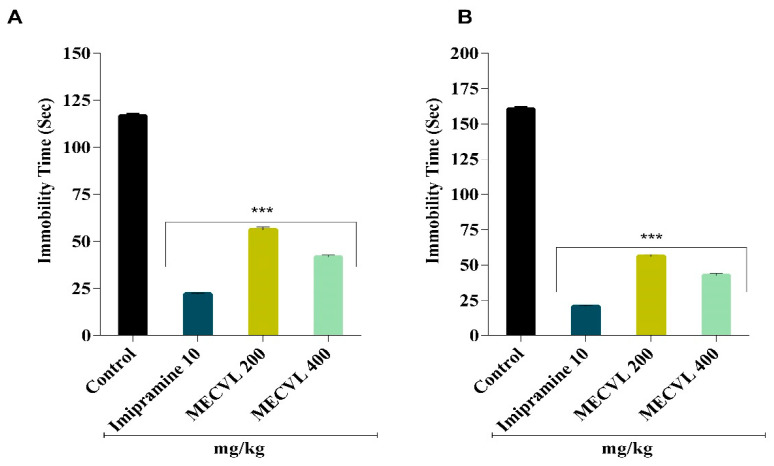
Effect of MECVL and imipramine in the force swimming test (**A**) and tail suspension test, (**B**) respectively. Each value is presented as the mean ± SEM (n = 5). *** *p* < 0.001 compared with the control group (Dunnett’s test); MECVL: Methanolic extract of *Chukrasia velutina* leaves.

After the oral administration of MECVL, statistically, the result obtained was from the force swimming test in Figure 2A. It portrayed that doses of 200 and 400 mg/kg showed a significantly (*p* < 0.001) anti-depressant effect by decreasing the immobile time when compared with the control group. As a reference drug, imipramine was used that also decreased the immobile time as the MECVL doses when compared with the control group.

#### 2.4.2. Effect of MECVL on Tail Suspension Test

In the tail suspension test, oral administration of MECVL at doses of 200 and 400 mg/kg into the rodents showed a significant (*p* < 0.001) anti-depressant effect through reducing the immobile time compared with the control group (Figure 2B). Imipramine was used as a reference drug that also reduced the immobile time such as MECVL doses when compared with the control group.

### 2.5. Anxiolytic Activity

#### 2.5.1. Effect of MECVL on Hole Board Test

**Figure 3:**
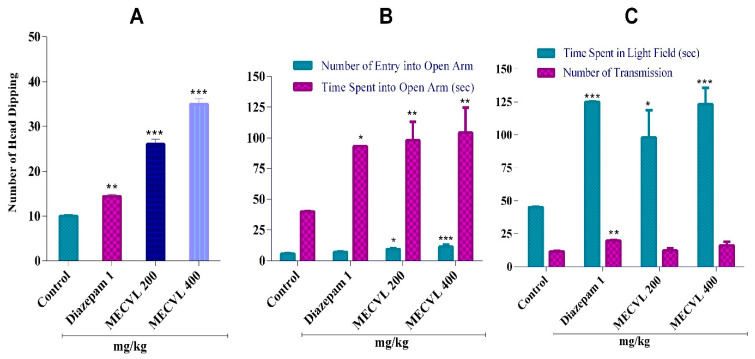
Effect of MECVL and diazepam in the hole board test (**A**), elevated plus maze test (**B**), and light/dark exploration test (**C**) in mice, respectively. Each value is presented as the mean ± SEM (n = 5). * *p* < 0.05, ** *p* < 0.01, and *** *p* < 0.001 compared with the control group (Dunnett’s test); MECVL: Methanolic extract of *Chukrasia velutina* leaves.

The result of the hole board test was reported in Figure 3A that showed a significant (*p* < 0.001) anxiolytic effect of both MECVL doses of 200 and 400 mg/kg through the number of head dipping into the hole of the test board. In doses of 200 and 400 mg/kg, the number of head dipping was increased (26.00 ± 1.14) and (35.00 ± 1.14) accordingly compared with the control. However, diazepam treated animals showed less number of head dipping (14.40 ± 0.51) compared to crude MECVL doses that were statistically significant (*p* < 0.01) compared with the control group.

#### 2.5.2. Effect of MECVL on Elevated Plus Maze Test

In an elevated plus maze test, the mice were treated with MECVL doses of 200 and 400 mg/kg which showed a significant anxiolytic activity (Figure 3B) through time spent in an open arm and the number of entries into open arm, respectively. In EPM, a MECVL dose of 400 mg/kg showed an increase in the number of entries into open arm which was statistically significant (*p* < 0.001) and also increased the time spent in the open arm that was also statistically significantly (*p* < 0.01) when compared with the control. Another MECVL dose of 200 mg/kg showed enhancement of the number of entries into the open arm that was significant (*p* < 0.05) and also increased the time spent into the open arm which was also significant (*p* < 0.01) when compared with the control. However, we can observe that the reference drug diazepam 1 mg/kg could not be able to show significance as the MECVL doses when it was compared with the control.

#### 2.5.3. Effect of MECVL on Light/Dark Box Test

The result of the light/dark box test is summarized in Figure 3C where we observed the rodent treated with diazepam (1 mg/kg) and increased the time spent in a light field and the number of transmissions that showed a significant (*p* < 0.001) anxiolytic activity compared with the control. In the LDB test, a MECVL dose of 400 mg/kg showed a significant (*p* < 0.001) increase in the time spent in a light field and a dose of 200 mg/kg showed a mild significant (*p* < 0.05) increase in the time spent in a light field when compared with the control.

### 2.6. Sedative Activity 

#### 2.6.1. Effect of MECVL on Hole Cross Test

The treatment of MECVL doses of 200 and 400 mg/kg and diazepam (1 mg/kg) produced the reduction of movement of mice in the hole cross test compared with the control in Table 2. In HCT, the movement of the rodent was observed from 0 to 120 min when diazepam showed a statistically significant (*p* < 0.001) sedation activity in mice when compared with the control. In addition, a MECVL dose of 400 mg/kg showed statistically significant (*p* < 0.001) locomotion activity during 30 to 120 min; however, a MECVL dose of 200 showed significant (*p* < 0.01) reduction of movement only in 90 min when compared with the control.

#### 2.6.2. Effect of MECVL on Open Field Test

The rodent was treated by MECVL doses of 200 and 400 mg/kg and diazepam (1 mg/kg) by reducing the number of squares crossed in the open field test and the result is summarized in Table 3. We observed the experiment during 0 to 120 min where diazepam showed a reduction in the number of squares crossed that was a statistically significant (*p* < 0.001) sedative activity from 30 to 120 min compared with the control. Furthermore, a MECVL dose of 400 mg/kg showed a statistically significant (*p* < 0.001) sedative activity in 90 and 120 min whereas a MECVL dose of 200 mg/kg showed significant (*p* < 0.001) sedative action only in 120 min through reducing the number of squares crossed when compared with the control group.

### 2.7. In Silico Studies: Pharmacokinetic Parameter Analysis by SwissADME

Lipinski’s rule of five, the SwissADME online tool was used for calculating the pharmacokinetics properties of the chemical compounds which are identified through GC-MS analysis. Lipinski’s rule stated that oral drugs/chemicals were bioavailable only when those drugs or chemicals are maintained by the following rules. These are: Molecular weight of the compound should be less than 500 amu, hydrogen bond acceptor sites should be less than 10, hydrogen bond donor sites should be less than 5, molecular refractivity among 40 to 130, and lipophilicity value < 5. By this calculation, we found 35 chemical compounds in Table 4 that followed Lipinski’s rule of five among these 57 compounds found through the GC-MS analysis.

### 2.8. Molecular Docking Study for Antidepressant Activity

In the study of antidepressant docking, thirty-five chemical constituents which were maintained by Lipinski’s rule of five from fifty-seven GC-MS analysis identified compounds were picked up. These compounds were docked against the human serotonin receptor (PDB: 5I6X) for exhibiting the range of docking scores from +4.354 to −5.18 kcal/mol. The result of the docking score was exhibited in Table 5. The result observed was that the compound 1,2,4-benzenetriol (−5.18 kcal/mol) evaluated the highest docking score and 13-tetradece-11-yn-1-ol (+4.354 kcal/mol) showed the lowest docking score against the selected receptor. All other compounds were docked against these selected enzyme receptors except diethyl mercaptal of d-mannose; dl-Allo-cystathionine; β-D-Glucopyranose, 1,6-anhydro-; germacrene D; cis-muurola-3,5-diene; β-copaene; quinic acid; 1-Heptanol, 2,4-dimethyl-, (*R*,*R*)-(+)-; d-Mannitol, 1-decylsulfonyl-; d-Mannitol, 1-thiohexyl-1-deoxy-; chlorozotocin; sparsomycin; 9-Dodecen-1-ol, acetate, (*Z*)-; cis-7-Tetradecen-1-ol; and 1,5-Hexadien-3-ol, trifluoroacetate. The antidepressant docking score of other compounds were as follows: 1,2,4 Benzenetriol > Phloroglucinol > 4-Diazodamantanone > Levomenthol > 5-Butyl-1,3-oxathiolan-2-one > 3-Methyl-2-furoic acid > D-Allose > Acetoacetic acid, 1,3-dithio-, *S*-ethyl ester > Dimethylmuconic acid > 2,4-Octadienoic acid, 7-hydroxy-6-methyl > 3-Nonyn-2-ol > Glycerol 1-palmitate > Tridecanoic acid, 12-methyl-, methyl ester > 3-Chloropropionic acid, 10-undecenyl ester > Undecanal > Butanoic acid, octyl ester > Decanal > Dodecanoic acid, 3-hydroxy- > Dodecanal > 13-Tetradece-11-yn-1-ol. Imipramine hydrochloride which was used as a reference standard drug exhibited the docking score of −5.35 kcal/mol against the human serotonin receptor (PDB: 5I6X) and it was attached with the same receptor through one hydrogen bond of Tyr 171 and five hydrophobic interactions of Val 479 (three interactions), Leu 492, and Ile 581. The docking analysis of the few highest score compounds expressed different binding interactions between the targeted receptor and the ligands in Figure S1. 1,2,4-Benzentriol interacted with the human serotonin receptor (PDB: 5I6X) through three hydrogen bonds of Gyl 476 (two interactions) and Ser 174 and two hydrophobic interactions with Leu 492 and Val 479 (docking score −5.18 kcal/mol). Phloroglucinol interacted with 5I6X through the composition of two hydrogen bonds of Ser 174 and Gly 476 residues and two hydrophobic interactions of Val 429 and Tyr 171 residues (docking score −4.741 kcal/mol). 4-Diazodamantanone interacted with the same protein receptor through the formation of one hydrogen bond Tyr 171 and two hydrophobic interactions Val 479 and Leu 492 (docking score −4.171 kcal/mol). Levomenthol was enclosed with the same protein receptor by nine hydrophobic interactions of Val 429, Val 488 (two interactions), Leu 245, Leu 577 (two interactions), Trp 573 (two interactions), and there was no hydrogen bond (docking score -3.911 kcal/mol). 5-Butyl-1,3-oxathiolan-2-one interacted with this similar protein through one hydrogen bond Tyr 171 and five hydrophobic bond interactions of Leu 245, Leu 248, Val 479, Val 488, and Tyr 171 (docking score −3.831 kcal/mol).

### 2.9. Molecular Docking Study for Anxiolytic Activity

In the evaluation of anxiolytic docking, the same thirty-five compounds were used which were previously docked against the human serotonin receptor for an antidepressant study. In this time, these compounds docked against the potassium channel receptor (PDB: 4UUJ) and the range of docking scores were +4.228 to −5.955 kcal/mol and the result is exhibited in Table 5. All of these compounds were docking against this receptor except diethyl mercaptal of d-mannose; dl-Allo-cystathionine; germacrene D; *cis*-muurola-3,5-diene; β-copaene; 1-Heptanol, 2,4-dimethyl-, (*R*,*R*)-(+)-; d-Mannitol, 1-decylsulfonyl-; d-Mannitol, 1-thiohexyl-1-deoxy-; chlorozotocin; sparsomycin; 9-Dodecen-1-ol, acetate, (*Z*)-; cis-7-Tetradecen-1-ol; 1,5-Hexadien-3-ol; and trifluoroacetate. The compounds which were docking against the potassium channel receptor were as follows: Phloroglucinol > 1,2,4 Benzenetriol > β-D-Glucopyranose, 1,6-anhydro- > Levomenthol > Quinic acid > 3-Methyl-2-furoic acid > 5-Butyl-1,3-oxathiolan-2-one > 4-Diazodamantanone > D-Allose > Dimethylmuconic acid > 2,4-Octadienoic acid, 7-hydroxy-6-methyl > Acetoacetic acid, 1,3-dithio-, *S*-ethyl ester > 3-Nonyn-2-ol > Glycerol 1-palmitate > Tridecanoic acid, 12-methyl-, methyl ester > Butanoic acid, octyl ester > 3-Chloropropionic acid, 10-undecenyl ester > Dodecanal > Dodecanoic acid, 3-hydroxy- > Decanal > Undecanal > 13-Tetradece-11-yn-1-ol. These compounds were compared with a reference standard drug diazepam and the docking score was −4.035 kcal/mol against the potassium channel receptor and it was attached with this receptor through eight hydrophobic bond interactions of Trp 163 (four interactions), Asp 165, Thr 164, Lys 142, Asp 143, and H-bond was not present. In the analysis, a few highest docking score compounds exhibited several binding interactions between the target protein and ligands in Figure S2. Phloroglucinol interacted with the potassium channel receptor with three hydrogen bonds of Glu 71, Thr 72, Gly 79, and no hydrophobic interaction occurred in this active site (docking score –5.955 kcal/mol). 1,2,4 Benzenetriol was enclosed with the same receptor through three hydrogen bonds of Thr 72, Gly 79, Leu 81, and one hydrophobic bond interaction of Gly 79 (docking score −5.771 kcal/mol). Then, β-D-Glucopyranose, 1,6-anhydro- interacted with a similar protein by three hydrogen bonds of Trp 68, Thr 72, Leu 81, and two hydrophobic interactions of Gly 77 and Gly 79 (docking score −4.675 kcal/mol). Levomenthol interacted with the same protein through one hydrogen bond of Leu 81 and nine hydrophobic bond interactions of Met 96 (two interactions), Ala 92, Pro 83, Trp 68 (two interactions), Tyr 82 (two interactions), Gly 77 (docking score −4.647 kcal/mol). Quinic acid was enclosed with the same receptor through two H-bond interactions of Trp 68, Gly 77, and four hydrophobic bond interactions of Gly 77 (two interactions), Pro 83, and Tyr 82 (docking score −4.42 kcal/mol).

### 2.10. Molecular Docking Study for Sedative Activity

In the study of sedative activity, the previously discussed thirty-five molecules were docking for the antidepressant and anxiolytic effect and these compounds were docking against the crystal structure of the human gabaa receptor (PDB: 4COF) for sedative action. Those compounds which followed Lipinski’s rule were docking against the crystal structure of the human gabaa receptor except the following: Diethyl mercaptal of d-mannose; dl-Allo-cystathionine; germacrene D; cis-muurola-3,5-diene; β-copaene; 1-Heptanol, 2,4-dimethyl-, (*R*,*R*)-(+)-; d-Mannitol, 1-decylsulfonyl-; chlorozotocin; sparsomycin; 9-Dodecen-1-ol, acetate, (*Z*)-; cis-7-Tetradecen-1-ol; and 1,5-Hexadien-3-ol, trifluoroacetate. The molecules were docked against the crystal structure of the human gabaa receptor which exhibited the docking score in Table 5 and the range was +4.67 to −6.942 kcal/mol. The compounds which were docking against this receptor were as follows: Quinic acid > Phloroglucinol > β-D-Glucopyranose, 1,6-anhydro- > 3-Methyl-2-furoic acid > 1,2,4 Benzenetriol > D-Allose > Dimethylmuconic acid > Levomenthol > Acetoacetic acid, 1,3-dithio-, *S*-ethyl ester > 5-Butyl-1,3-oxathiolan-2-one > 4-Diazodamantanone > 2,4-Octadienoic acid, 7-hydroxy-6-methyl > Glycerol 1-palmitate > 3-Nonyn-2-ol > d-Mannitol, 1-thiohexyl-1-deoxy- > Dodecanoic acid, 3-hydroxy- > Tridecanoic acid, 12-methyl-, methyl ester > 3-Chloropropionic acid, 10-undecenyl ester > Undecanal > Butanoic acid, octyl ester > Decanal > Dodecanal > 13-Tetradece-11-yn-1-ol. Diazepam was used as a reference standard drug which was docking against the crystal structure human gabaa receptor through eight hydrophobic bond interactions of Tyr 157 (three interactions), Tyr 205, Ala 201, Asp 43, Tyr 62, and Phe 200 but no H-bond interaction and the docking score was −5.961 kcal/mol. A few compounds of docking scores were so close to the reference standard drug (Figure S3) and we mentioned them as follows: Quinic acid interacted with the crystal structure of the human gabaa receptor through six hydrogen bonds of Gln 64, Tyr 157, Tyr 205, Thr 202, Glu 155 (two interactions), and one hydrophobic bond interaction of Tyr 157 (docking score −6.942 kcal/mol). Phloroglucinol attached with the same receptor through four H-bond interactions of Gln 64, Thr 202, Glu 155, Tyr 97, and two hydrophobic bond interactions of Tyr 157 and Phe 200 (docking score −6.151 kcal/mol). Then, β-D-Glucopyranose and 1,6-anhydro were enclosed with a similar receptor through five hydrogen bonds of Gln 64, Thr 202, Glu 155, Tyr 97, Tyr 157, and three hydrophobic bond interactions of Glu 155 (two interactions) and Tyr 157 (docking score −5.895 kcal/mol). 3-Methyl-2-furoic acid was attached with the same receptor through two H-bonds of Trp 68 and Leu 81 and three hydrophobic bond interactions of Met 96, Gly 77, and Tyr 82 (docking score −5.608 kcal/mol). 1,2,4 Benzenetriol was enclosed with a similar receptor by three hydrogen bonds of Glu 155 (two interactions), Gln 64, and two hydrophobic bond interactions of Tyr 62 and Tyr 205 (docking score −5.447 kcal/mol).

### 2.11. Chemical Structures

**Figure 4:**
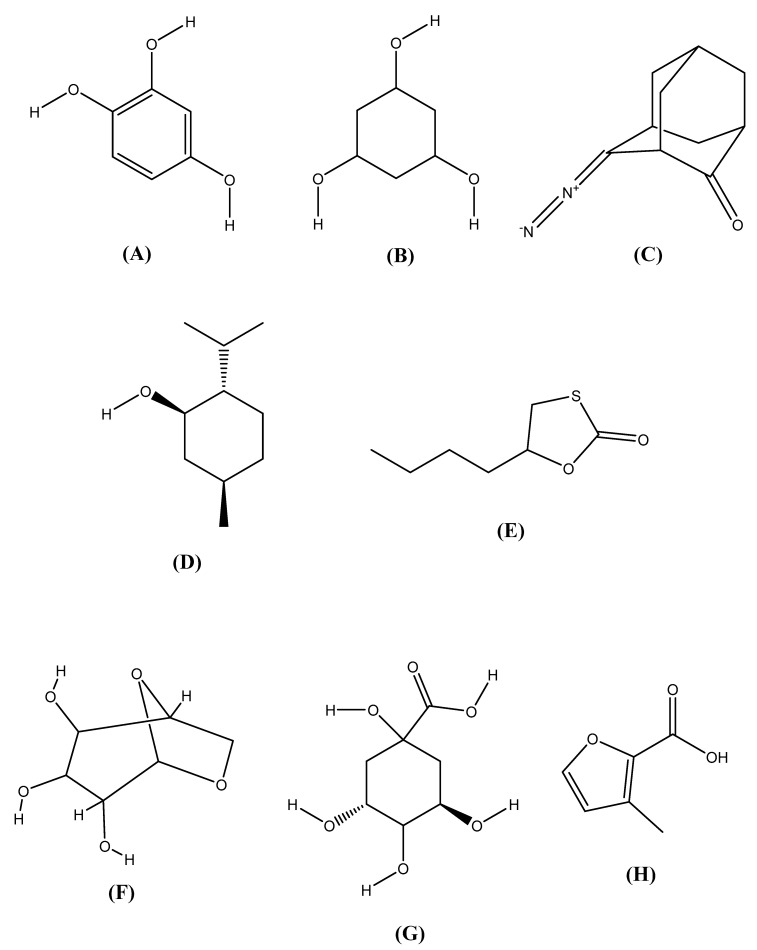
Chemical structure of (**A**) 1,2,4-Benzenetriol, (**B**) Phloroglucinol, (**C**) 4-Diazodamantanone, (**D**) Levomenthol, (**E**) 5-Butyl-1,3-oxathiolan-2-one 5, (**F**) β-D-Glucopyranose, 1,6-anhydro-, (**G**) Quinic Acid, and (**H**) 3-Methyl-2-furoic acid (structures were drawn using ChemDraw Professional 16.0).

The major bioactive compounds which have given the highest docking score are represented in Figure 4 and these compounds are 1,2,4 Benzenetriol; 3-Methyl-2-furoic acid; phloroglucinol; β-d-glucopyranose, 1,6-anhydro-; quinic acid; 4-Diazodamantanone; 5-Butyl-1,3-oxathiolan-2-one; and levomenthol.

## 3. Discussion

Medicinal plants are an affluent source of phytochemical compounds that can play a vital role to treat several chronic diseases [7]. An extensive number of potent biomolecules come from a diverse number of medicinal plants in recent times [2,18]. Scientists believed that these potent chemical constituents obtained from nature are used for treating many disorders with fewer side effects [19]. These potent compounds are highly capable of inhibiting the harmless act of a multiple number of chronic diseases [20]. Few chronic diseases are so critical and there are no specific drugs for those diseases [21]. In such cases, medicinal plants should be applied and they give an effective result in pharmacologically and phytochemically [22]. In the current study, we mainly showed that the effects of the methanolic extract of *Chukrasia velutina* leaves on the central nervous system [6] through multiple numbers of animal models. Depression and anxiety are those mental problems that divert mental health in many pathways such as insomnia, Alzheimer disease, etc. [3]. In all over the world, mental disorders are a severe problem nowadays and depression, anxiety, insomnia play a key role in improving mental disorders and behavioral diseases [23].

Neuropsychiatric disorders are one of the complex and numerous problems in current medical health issues. Parkinson’s disease, Alzheimer’s disease, anxiety, depression, insomnia, etc. are very crucially affected by the human health [24]. Medicinal plants are used to treat a lot of psychiatric disorders through their potential active phytochemicals [25] which have fewer side effects [26]. Since synthetic drugs reduce these disorders with a great number of side effects and sometimes they create addiction over these drugs which causes suicide [27]. To develop a potential neuropsychiatric drug with a tolerable number of side effects [28], we used several animal models for testing medicinal plants chemical constituents’ efficacy by following phytochemical and pharmacological analysis [29].

Numerous multiple animal models were used for showing the antidepressant efficacy of MECVL; however, the force swimming test (FST) and tail suspension test (TST) in rodents were the most reliable tests for identifying the antidepressant action of plants [11] because these tests were efficient as a remarkable system to wedge the depressing ambiance in rodents [3]. In these two tests, we usually showed the immobile time of the animal when these were recorded in swimming and suspending conditions, respectively [5]. The immobile time indicates that the animal should be hopeless and feeling numb. When the time of immobility should be reduced that means the sample has an antidepressant activity that causes a reduction of immobile time. The present study disclosed that the immobile time of MECVL (200 and 400 mg/kg) should be decreased compared with the control immobile time which was significant (*p* < 0.001). Imipramine was used as a standard drug in both FST and TST and it also exhibited less immobile time compared with the control [30]. In both FST and TST, we explored that the immobile time of MECVL (200 and 400 mg/kg) and immobile time of the reference drug imipramine exhibited almost similar efficacy and these were significant (*p* < 0.001). 

Depression creates anxiety inside of the mind and the wired behavior happens [12]. To investigate the anxiolytic action of MECVL, we selected a few animal models such as the hole board test, elevated plus maze test, and light/dark box test in rodents and these models are very classic and standard for determining the anxiolytic effect of plant extract [8,31,32]. To consider a hypothesis of HBT, the head dipping of rodents is conversely proportional to their state of anxiety in the tolerable aversive condition [6,10]. Therefore, the enhancement of the number of head dipping into the hole of the board represents the attenuate anxiety state [11]. In HBT, diazepam was used as a reference drug and it exhibited that the number of head dips into the board was significant (*p* < 0.01) when it was compared with the control. The current study stated that after oral administration of several doses of MECVL (200 and 400 mg/kg), the number of head dips into the board increased into the rodents when compared with the control and these doses were significant (*p* < 0.001) against anxiety. Moreover, EPM is a much-anatomized method for investigating the anxiolytic activity of MECVL [24]. In this method, the increment number of entries and time spent in the open arms was applied for the evaluation of the anxiolytic effect [10]. Diazepam was used as a standard drug that exhibited an increment in the time spent into an open arm when compared with the control and it was significant (*p* < 0.05). However, the reference drug did not show any significance over the number of entries into the open arm [33]. On the other hand, the doses of MECVL (200 and 400 mg/kg) showed that the number of entries and time spent into both open arms were rising when compared with the control. Especially the dose of 400 mg/kg of MECVL asserted that the number of entries into open arm increased when compared with the control and it was significant (*p* < 0.001). In addition, the same dose of MECVL showed a significant (*p* < 0.01) according to the time spent in the open field when compared with the control. Similar to EPM, the LDB test is also a standard model for assessing the anxiolytic effects. In the LDB test, it works on the implicit reluctance of rodents when entering into the bright field and natural empiric behavior of rodents in response to the ideal condition and light. The reference drug diazepam was used which exhibited significant action against anxiety both in the time spent in the illuminate area and the number of transmissions [10]. However, in the present study, we found that multiple doses of MECVL (200 and 400 mg/kg) displayed significant rising of the time spent in the light box but there was no significant action in the number of transmission of rodents into the hole of the box.

Insomnia depends on many key factors such as stress, anxiety, depression, etc. [31]. Bioactive compounds sometimes play a crucial role in inhibiting insomnia problems because insomnia leads the human being to a lot of chronic diseases such as hypertension, diabetics, heart disorder, cancer, etc. [9]. In the present study, we exhibited that MECVL can play a crucial role in insomnia by reducing anxiety and depression through versatile mice models [34]. By using these animal models (open field and hole cross tests) we can determine how MECVL acts on rodents for sedative action [35]. The effect of sedation was mainly dose-dependent for MECVL. Two doses of MECVL were significantly decreasing the movement of the animal in both test models. The hole cross test (HCT) and open field test (OFT) are very standard and classic models to determine the sedative action of the extract. In HCT, the rodents reduced their movement with time [36]. In this test, the observation was highlighted mainly on the dose of 400 mg/kg and its first (0 min) to fifth (120 min) observation, the number of the crossing of rodent into the hole of the wall decreased when the time was passed because it was observed at 30 min duration of time [37]. However, the 200 mg/kg dose of MECVL showed a significant reduction in the number of crossing between the hole of partition only for the fourth observation [38]. In addition, diazepam which was used as a reference drug exhibited significant sedative activity for its observation from 0 to 120 min. By this overview, the 400 mg/kg dose of MECVL showed a significant sedative activity for all its observation similar to the reference drug diazepam when all of them were compared with the control [31]. Similar to HCT, OFT is also a transcendent method for testing the sedation effect [33]. In this test, the animal crossed the box with white and black color and the sedative activity was assessed [39]. In OFT, the doses of MECVL showed the CNS depressant effect which creates sedation in the rodent body and the 400 mg/kg dose of MECVL asserted a significant sedative activity in the fourth (90 min) and fifth (120 min) observation. However, the 200 mg/kg dose of MECVL showed a significant sedation action only for the fifth (120 min) observation [21]. Furthermore, diazepam was used as a reference drug that exhibited a significant sedative action from the second (30 min) to fifth (120 min) observation. Therefore, the 400 mg/kg dose of MECVL acted as nearly less than the reference drugs than the 200 mg/kg dose when all were compared with the control [40].

The mechanisms of neuropsychiatric disorders are usually the result of either an imbalance of neurotransmitters such as serotonin, GABA, and dopamine or abnormal transmission of serotonergic, glutamatergic, noradrenergic, and GABA-ergic [2,41]. Bioactive compounds are obtained from medicinal plants that should be regulated and inhibit the abnormal release of neurotransmitters [42]. Additionally, in the secondary metabolites of *C. velutina* leaves, we tested a few qualitative phytochemical screenings where we found some chemical compounds such as resin, phenol, tannin, alkaloid, carbohydrate, and glycosides which played a great role as a psychiatric drug [22,25]. In our study, we evaluated MECVL as a neuropsychiatric drug through several animal models against depression, anxiety, and insomnia where the phytochemicals such as resin, phenol, tannin, alkaloid, carbohydrate, and glycoside played a significant role by binding with protein complexes. We also found a diverse number of phytocompounds through the GC-MS analysis which were a great impact on the receptors that worked in multiple neurological diseases such as human serotonin receptor, potassium channel receptor, the crystal structure of human gabaa receptor, etc. [2]. The nature of these bioactive compounds are mostly polyphenol or alcohol-based. For these reasons, most of the phytoconstituents are active against several microorganisms as we knew from the literature review in Table 6. Mostly severe kinds of neurobiological diseases were caused by infectious microorganisms [43]. However, these compounds were active against neurobiological disorders as infectious organisms whereas psychiatric disorders are also responsible for oxidative stress [44].

Molecular docking is an exigent part of in silico approaches and also an important tool in structural molecular biology and computer assisted-drug design (CADD) [97]. This tool is used for predicting the molecular mechanism and pharmacological action of the compounds which is found from medicinal plants [98]. The pharmacokinetics parameter analysis by SwissADME is also a part of in silico approaches. In this study, we found fifty-seven compounds in *C. velutina* through the quantitative GC-MS analysis, and these compounds were analyzed through the SwissADME tool for ADME/T analysis [99,100]. By analyzing these compounds, we found only thirty-five compounds of *C. velutina* which followed Lipinski’s rule in the SwissADME tool with a molecular weight not more than 500; H-bond acceptors ≤ 10; H-bond donors ≤ 5; molar refractivity among 40 to 130; lipophilicity < 5. Therefore, these compounds maintained pharmacokinetics parameters indicating that these compounds do not have any toxic impact in the body [38]. After that, these compounds should be used in the molecular docking analysis for antidepressant, anxiolytic, and sedative effects to correlate the experiment results that we found through the animal model and these compounds have been exhibited in Table 5.

In the antidepressant docking analysis, we showed that only twenty compounds were docked against the human serotonin receptor (PDB: 5I6X) among those thirty-five compounds in Table 5, which were selected for the docking of antidepressant action [2,101]. These compounds are 1,2,4 Benzenetriol > Phloroglucinol > 4-Diazodamantanone > Levomenthol > 5-Butyl-1,3-oxathiolan-2-one > 3-Methyl-2-furoic acid > D-Allose > Acetoacetic acid, 1,3-dithio-, *S*-ethyl ester > Dimethylmuconic acid > 2,4-Octadienoic acid, 7-hydroxy-6-methyl > 3-Nonyn-2-ol >Glycerol 1-palmitate > Tridecanoic acid, 12-methyl-, methyl ester > 3-Chloropropionic acid, 10-undecenyl ester > Undecanal >Butanoic acid, octyl ester > Decanal > Dodecanoic acid, 3-hydroxy- > Dodecanal > 13-Tetradece-11-yn-1-ol. In this analysis, imipramine was used as a reference standard drug and it was attached with the human serotonin receptor and gave a docking score of −5.35 kcal/mol, and a few compounds among these twenty constituents were exhibited a closed docking score considered to the reference standard drug when they were enclosed with the human serotonin receptor for the antidepressant effect [30]. The compounds which were close to the imipramine such as 1,2,4 Benzenetriol (docking score −5.18 kcal/mol), Phloroglucinol (docking score −4.741 kcal/mol), 4-Diazodamantanone (docking score −4.171 kcal/mol), and Levomenthol (docking score −3.911 kcal/mol), 5-Butyl-1,3-oxathiolan-2-one (docking score −3.831 kcal/mol) have manifested an excellent docking score. Among these compounds, 1,2,4 Benzenetriol is the one that exhibited the highest docking score against the human serotonin receptor (PDB; 5I6X) and was most close to the reference standard drug.

In the anxiolytic docking analysis, twenty-two compounds among the thirty-five compounds were docking against the potassium channel receptor (PDB: 4UUJ) for anxiolytic activity in Table 5. These compounds are phloroglucinol > 1,2,4 Benzenetriol > β-D-Glucopyranose, 1,6-anhydro- > Levomenthol > Quinic acid > 3-Methyl-2-furoic acid > 5-Butyl-1,3-oxathiolan-2-one > 4-Diazodamantanone > D-Allose > Dimethylmuconic acid > 2,4-Octadienoic acid, 7-hydroxy-6-methyl > Acetoacetic acid, 1,3-dithio-, *S*-ethyl ester >3-Nonyn-2-ol > Glycerol 1-palmitate > Tridecanoic acid, 12-methyl-, methyl ester > Butanoic acid, octyl ester > 3-Chloropropionic acid, 10-undecenyl ester >Dodecanal > Dodecanoic acid, 3-hydroxy- > Decanal > Undecanal > 13-Tetradece-11-yn-1-ol Diazepam were used as a reference standard drug which interacted with the same receptor and gave a docking score of −4.035 kcal/mol for the anxiolytic activity. A few docking score compounds such as Phloroglucinol (docking score −5.955 kcal/mol); 1,2,4 Benzenetriol (docking score −5.771 kcal/mol); β-D-Glucopyranose, 1,6-anhydro- (docking score −4.675 kcal/mol); levomenthol (docking score −4.647 kcal/mol); and Quinic acid (docking score −4.42 kcal/mol) were very close to the reference standard drug docking score when they were attached to the potassium channel receptor. Among these phytochemicals phloroglucinol is the one that has an excellent docking score and it is very similar to diazepam.

In the analysis of sedative docking, we observed that twenty-three compounds between the thirty-five compounds of this MECVL were docking against the crystal structure of the human betaa receptor (PDB: 4COF) for sedative activity in Table 5. These compounds are Quinic acid >Phloroglucinol > β-D-Glucopyranose, 1,6-anhydro- > 3-Methyl-2-furoic acid > 1,2,4 Benzenetriol > D-Allose > Dimethylmuconic acid > Levomenthol > Acetoacetic acid, 1,3-dithio-, *S*-ethyl ester > 5-Butyl-1,3-oxathiolan-2-one > 4-Diazodamantanone > 2,4-Octadienoic acid, 7-hydroxy-6-methyl > Glycerol 1-palmitate > 3-Nonyn-2-ol > d-Mannitol, 1-thiohexyl-1-deoxy- > Dodecanoic acid, 3-hydroxy- > Tridecanoic acid, 12-methyl-, methyl ester > 3-Chloropropionic acid, 10-undecenyl ester >Undecanal > Butanoic acid, octyl ester > Decanal > Dodecanal > 13-Tetradece-11-yn-1-ol. The reference standard drug was used as diazepam which was enclosed with this receptor and delivered a docking score of −5.961 kcal/mol. Several numbers of the compounds exhibited the docking scores which were close to the docking score of diazepam and all of these were attached with a similar receptor. Quinic acid, phloroglucinol, and β-D-Glucopyranose, 1,6-anhydro- are those compounds that have exhibited a high docking score and these are the most nearest to the reference standard drug. Antidepressant, anxiolytic, and sedative effects of *C. velutina* might be explained through the presence of these compounds which showed a good binding affinity for all receptors and thus these compounds might be responsive for the aforementioned pharmacological activities [8]. From all these results, we can decide that the studied phytochemicals such as 1,2,4 Benzenetriol, phloroglucinol, quinic acid, β-D-Glucopyranose, 1,6-anhydro-, and levomenthol have exhibited a great role in antidepressant, anxiolytic, and sedative effects of secondary metabolites of *C. velutina* leaves (MECVL). In the previous studies of these molecules which have the highest docking score, we showed that 1,2,4 benzenetriol, phloroglucinol, quinic acid, β-D-Glucopyranose, 1,6-anhydro-, levomenthol, 4-Diazodamantanone, 5-Butyl-1,3-oxathiolan-2-one, 3-Methyl-2-furoic acid, as well as their chemical structure in Figure 4 and all of these have a common effect as exhibited in Table 6. Additionally, these are antioxidant [67], anti-inflammatory [88] and antimicrobial [51] effects that are also responsible for neuropsychiatric diseases [102]. Therefore, these compounds are potential and bioactive against these neuropsychiatric diseases such as depression, anxiety, insomnia, etc.

The major bioactive phytocompounds which are found from the docking analysis have several biological activities that are represented in Table 6. A number of compounds are responsible for the neuro-pharmacological activities that were discussed in the docking study where most of the compounds have shown an excellent binding affinity with the respective receptors that are responsible for the neuropsychiatric disorders. These bioactive compounds also showed several pharmacological actions such as anti-inflammatory, antioxidant, anti-microbial, anti-nociceptive, and anti-tumor activities. Benzenetriol has showed a great binding affinity against the receptors which is responsible for anti-depressant, anxiolytic, and sedative effects, respectively. This compound also has an antimicrobial activity as shown in Table 6. 3-Methyl-2-furoic acid, this molecule has a strong binding affinity against the receptor which is responsible for the sedative effect. 3-Methyl-2-furoic acid has a good biological property as anti-fungal and anti-tumor activities and these biological properties can play a crucial role against insomnia. Phloroglucinol is one of the compounds that has a great binding affinity against all of the three receptors which is responsible for anti-depressant, anxiolytic, and sedative effects, respectively and it has also several pharmacological actions such as antioxidant, antineoplastic, anti-inflammatory, anti-microbial, and anti-fungal activities. The compound (β-D-Glucopyranose, 1,6-anhydro-) has a good docking score against the receptor which is responsible for anxiolytic and sedative effects and its biological actions are antioxidant and anti-bacterial effects and we know that oxidation and microorganisms produce many neuropsychiatric problems. D-Allose exhibited a good docking score against the receptor and possesses several biological activities such as antioxidant, anti-bacterial, and anti-viral effects. Quinic acid exhibited an effective docking score against the receptors which is responsible for anxiolytic and sedative effects and it also has several numbers of biological activities such as anti-bacterial, anti-viral, antioxidant, and hepatoprotective effects. 4-Diazodamantanone has shown a binding affinity against the receptor which is responsible for depression and its biological activities are anti-inflammatory, antimicrobial, and anticholinesterase effects. Levomenthol showed a great binding affinity with the receptors that causes depression and anxiety and it has a good number of biological actions such as antimicrobial, nematocidal, cytotoxic, anti-influenza, and inflammation-promoting activities, antioxidant effects. 5-Butyl-1,3-oxathiolan-2-one has a good docking score against the receptor which creates depression and also has a biological properties such as the antiviral effect.

## 4. Materials and Methods

### 4.1. Drugs and Chemicals

The source of the chemicals is given here: Methanol (Merck Darmstadt, Germany), DMSO, Tween-80 (Sigma–Aldrich, St. Louis, MO, USA), imipramine, and diazepam (Square Pharmaceuticals Ltd.).

### 4.2. Plant Collection, Identification, and Extract Preparation

The leaves of *C. velutina* were assembled from the forest of Bangladesh Forest Research Institute, Chittagong, Bangladesh, in April 2019 and identified by the expert of Bangladesh Forest Research Institute (BFRI). A specimen voucher (BFRI SR0918) was deposited at the BFRI, Bangladesh for further reference. Then, the leaves were left to dry into the air at room temperature and ground into fine powder by the electric grinder (Super Turbo Dix: Mix-104A) from Groupe SEB India (P) Ltd. in Himachal Pradesh, India. A 500 g of leaves powder was soaked in 1500 mL of methanol. After 10 days, it was filtered with cotton and Whitman Filter Paper number 1. Further, the solvent of methanol was evaporated in a water bath (Model: SM WB 5 LITER, Samarath Electronics, Thane, Maharashtra, India) at 60–65 °C temperature and 18.27 g of methanolic extract of *C. velutina* leaves (MECVL) was obtained, it was filled in a glass vial and stored in the refrigerator at 4 °C temperature. This crude extract was used for the evaluation of antidepressant, anxiolytic, and sedative actions.

### 4.3. Qualitative Phytochemical Screening of MECVL

A small number of recently prepared methanolic extract of *C. velutina* leaves was partly subjected to an initial qualitative phytochemical evaluation for the detection of phytochemicals such as resin, carbohydrates, phenol, tannin, alkaloid, saponins, flavonoid, glycoside, steroid, cholesterol, polyphenol, lencoanthocyanin, etc. by using the following standard methods which were reported previously by Tiwari et al. [103].

### 4.4. GC-MS Analysis of MECVL

The GC-MS analysis of methanolic extract of *C. velutina* leaves (MECVL) was assessed by employing Agilent (7890A) Technologies capillary gas chromatography along with a mass spectrometer. The column exerted was an absorbed silica capillary column of 95% dimethyl-poly-siloxane and 5% phenyl (HP-5MSI; length: 90 m, diameter: 0.250 mm and film: 0.25 µm). A 250 °C of injector temperature, fontal oven temperature at 90 °C gently elevated to 200 °C at a speed of 3 °C/min for 2 min and a final increase to 280 °C at 15 °C/min for 2 min, and these were the parameters for GC-MS detection. The total GC-MS run time was 36 min, with Helium 99.999% as carrier gas, used at a column flow rate of 1 mL/min. The fixed interface temperature of GC to MS was 280 °C and the MS in scan mood was set on an electron ionization system. MS quad and source temperature were compiled at 150 and 230 °C, respectively where the mass range investigated was 50–550 *m*/*z*. Each component was used to search and identified by the “NIST-MS Library 2009”. Measurement of the relative percentage amounts of each compound was determined using the peak area expression of the “TIC” (total ionic chromatogram), with calculations being done automatically.

### 4.5. Animals and Ethical Statements

Swiss albino mice were collected from Jahangir Nagar University, Savar, Dhaka, Bangladesh. These mice were male and weighing about 20–25 gm. Animals were kept in polypropylene cages by maintaining a suitable laboratory condition where the room temperature was 25 ± 2 °C and relative humidity was 55–60%; 12 h light/dark cycle with a standard laboratory food ad libitum. All of the experiments with mice occurred in peaceful conditions and the animal adapted the laboratory condition before 10 days of the experiment. These studies were held following the internationally accepted principle for appropriate use of laboratory animals, namely the National Institutes of Health (NIH) and International Council for Laboratory Animal Science (ICLAS). The current study procedures were reviewed and approved by the “P&D committee” of the Department of Pharmacy, International Islamic University Chittagong, Bangladesh, with reference number Pharm-P&D-80/06’19-151.

### 4.6. Acute Toxicity Testing of MECVL

The acute toxicity test was executed by using standard laboratory conditions following the “Organization for Environmental Control Development” (OECD) guidelines. The experimental animals (n = 5) of the control and test sample of MECVL groups were administered orally to each group with the control (1% Tween-80 solutions) or a single test sample dose (5, 10, 100, 200, 400, 1000, and 2000 mg/kg) of the MECVL. Mice were not provided food and kept fasting overnight before oral administration and it should also be noticed that the mice could not be provided with any food about 3 to 4 h after oral administration of the extract [7].

### 4.7. Dosing Groups

In the present experiment, the mice were anonymously selected into four groups and every group had five mice (n = 5). Here, the control group received 1% Tween-80 solutions; fluoxetine and diazepam were considered as a reference drug which was used as a positive control (10 and 1 mg/kg, body weight), respectively; whereas the residual groups of MECVL were given 200 and 400 mg/kg body weight of the rodent.

### 4.8. Antidepressant Activity of MECVL

#### 4.8.1. Force Swimming Test

The forced swimming test is commonly used in experiments of antidepressant-like activity in rodents. Treatment with the MECVL was conducted by oral administration of 200 and 400 mg/kg. The reference drug, imipramine 10 mg/kg was administered intraperitoneally and Tween-80. The negative control was administered orally at 10 mL/kg of body weight of the rodent. Each mouse was individually placed in an open cylindrical glass container (45 cm height × 20 cm diameter) containing 17 cm of water at 25 °C for 5 min. An immobile time was recorded for each mouse when it was floating motionless or creating only those movements needed to keep its head above water.

#### 4.8.2. Tail Suspension Test

This behavior portrayed in rodents that inhibited to imminent and fateful stresses during the tail suspension assay were considered behavioral despair, which is imagined depression in humans. Treatment with the MECVL was conducted by oral administration of 200 and 400 mg/kg. The reference drug, imipramine 10 mg/kg was administered intraperitoneally and Tween-80. The negative control was administered orally at 10 mL/kg of body weight of the rodent. Each mouse was hanged individually 50 cm above the floor using adhesive tape placed approximately 1 cm from the tip of their tails. The immobility time was recorded for 6 min and it was considered immobile when they silently hung or remained static.

### 4.9. Anxiolytic Activity Analysis of MECVL

#### 4.9.1. Hole Board Test

The hole board test was broadly used as an authentic pharmacological method for evaluating the anxiolytic or anxiogenic effect of rodents. The hole board is an apparatus that consists of a wooden box (40 × 40 × 25 cm) with sixteen equidistant holes (diameter 3 cm) evenly distributed on the base of the box. The apparatus was raised 25 cm above the floor. Each mouse was placed individually on the center of the board after 30 min of oral administration of treatments. The number of head dipping was counted in a period of 5 min.

#### 4.9.2. Elevated Plus Maze Test

The elevated plus maze assay is the test used to determine the anxiolytic activity of sample drug. A very commonly known elevated plus maze tool is used in this assay. Elevated plus maze consists of two opposing open arms (30 × 5 cm) and two closed arms (30 × 25 × 5 cm), likewise opposing cross-shaped arms. The open and closed arms are connected by a central platform (5 × 5) high and 45 cm from the floor. The animals were placed in the center of the apparatus with the head turned to towards one of the closed arms and their behavior was observed for 5 min. The behavioral measures were recorded: Number of entries in the open arms (NEOA) and length of stay of the animal in the open arms (LSOA).

#### 4.9.3. Light/Dark Box Test

The light/dark box test is conducted in an apparatus which is an open-topped rectangular box (46 × 27 × 30 cm^3^), divided into small (18 × 27 cm^2^) and large (27 × 27 cm^2^) compartments with a fixed partition containing a small hole (3 cm in diameter) in the middle. The small compartment was closed with a lid and painted in black color. On the other hand, the large compartment was painted in white color which was illuminated by an electric bulb (60 W) that was suspended on the top (120 cm above) of it. Mice were treated with Tween-80, MECVL, or diazepam and placed in the middle of the bright compartment. Then, the time spent by the animals in the bright compartment and a total number of transitions between the bright and dark compartments were recorded for 5 min.

### 4.10. Sedative Effect of MECVL

#### 4.10.1. Hole Cross Test

The hole cross test was conducted in a box which is a cage of (30 × 20 × 14 cm) in size with a partition of a wall in the middle having a hole of 3 cm in diameter. Mice were treated with 1% Tween-80, diazepam, and sample doses. Each animal was kept in one part of the box. Then, the total number of the crossing of an animal through the hole was counted for 3 min at 0, 30, 60, 90, and 120 min after the administration.

#### 4.10.2. Open Field Test

The open field test was assessed for sedative activity in mice. This apparatus consisted of a wooden square box (50 × 50 × 40 cm) with the floor divided into twenty-five small squares of equal dimensions (10 × 10) cm^2^ marked by white and black color. Mice were treated with 1% Tween 80, diazepam, and sample extract. Each mouse was placed individually at the center of the open field and observed for 3 min to record the number of squares crossed by a rodent with its four paws. The observation was counted as the number of squares crossed at 0, 30, 60, 90, and 120 min after the administration.

### 4.11. In Silico Molecular Docking

#### 4.11.1. Determination of Pharmacokinetic Parameters by SwissADME

The drug-likeness properties or pharmacokinetics parameters of all major compounds were identified by GC-MS and evaluated through SwissADME, an online tool (http://www.swissadme.ch/) by following Lipinski’s rule of five. Lipinski narrated that a chemical constituent could show drug-like activity if it does not default one of the following parameters: (i) Molecular weight not more than 500; (ii) H-bond acceptors ≤ 10; (iii) H-bond donors ≤ 5, (iv) molar refractivity among 40 to 130; and (v) lipophilicity < 5. Those constituents maintained Lipinski’s rule and they were considered as ideal drugs.

#### 4.11.2. Molecular Docking

The main pharmacologically active compounds of MECVL were identified by GC-MS analysis. These compounds were analyzed through SwissADME and selected those compounds that maintained the Lipinski’s rule of five. These specific compounds were selected for the molecular docking study, to assume a better endurable molecular interaction that emerged on their aptitude to interact with several target proteins. Docking studies were assessed by using Schrödinger Suite-Maestro v. 10.1, LLC, New York, NY, USA, and the Accelrys Discovery Studio 4.0 software (BIOVIA, San Diego, CA, USA) was assayed for visualization of 3D structures.

##### Ligand Preparation

The chemical structure of principle bioactive compounds of MECVL were downloaded from the PubChem compound repository (https://pubchem.ncbi.nlm.nih.gov/). LigPrep tool was utilized for ligand preparation which was adorned in Schrödinger Suite-Maestro v. 10.1 where several parameters were considered as follows: the OPLS_2005 force field and neutralized at pH 7.0 ± 2.0 using Epik 2.2 were used for minimization.

##### Receptor/Enzyme Preparation

The three-dimensional crystallographic structure of receptors/enzymes were obtained from the Protein Data Bank RCSB PDB [104]: Human serotonin receptor (PDB: 5I6X) [105], potassium channel receptor (PDB: 4UUJ) [106], and human gabaa receptor (PDB: 4COF) [107]. A docking experiment using the Protein Preparation Wizard was used for preparing the receptor/enzyme that was adorned in Schrödinger Suite-Maestro v. 10.1 as we discussed previously [2].

##### Glide Standard Precision Docking

Molecular docking analysis was performed to express the probable mechanism of action of the chosen constituents behind the neurological effects of the MECVL against the several receptors/enzymes for an antidepressant, anxiolytic, and sedative activity. Docking experiments were executed by using Glide standard precision docking that was adorned in Schrödinger Suite-Maestro v. 10.1 as we discussed previously [2,108].

## 5. Statistical Analysis

All of the data have been analyzed with SPSS version 20 (Statistical package for the social sciences) software (Schrodinger, LLC New York, NY, USA) by using one-way ANOVA followed by Dunnett’s test and values were expressed as the mean ± SEM (standard errors of means) where the *p*-value was considered less than 0.05 and it was statistically significant.

## 6. Conclusions

It can be summarized from the result of our study that the methanolic extract of *Chukrasia velutina* leaves (MECVL) has substantial and significant neuropharmacological effects such as antidepressant, anxiolytic, and sedative effects. These kinds of results might be due to the presence of several phytochemicals such as alkaloids, tannins, phenols, glycosides, and the abundance of major bioactive phytocompounds found during the GC-MS analysis which exerts their actions either individually or synergistically. Furthermore, our molecular docking study also demonstrated that eight bioactive phytocompounds namely 1,2,4-Benzenetriol, phloroglucinol, 4-Diazodamantanone, levomenthol, 5-Butyl-1,3-oxathiolan-2-one 5, β-D-Glucopyranose, 1,6-anhydro-, quinic acid, and 3-Methyl-2-furoic acid have the highest binding affinity towards the respective receptors which are responsible for the observed antidepressant, anxiolytic, and sedative properties. Additionally, from our ADME/T study, we found that these phytocompounds are safe from the draggable point of view though further extensive reviews regarding these compounds are still required to reveal their pharmacological activity in animal models. However, additional extensive studies are still necessary, for the most part on the isolation of the new bioactive compounds, and to explicate their detailed molecular mechanism of actions in animal models underlying the observed biological effects of the plant.

## Figures and Tables

**Table 1:** Quantitative compounds identified from MECVL through the GC-MS analysis.

S.N.	R.T. (min)	Compound Name	*m/z*	Area	Molecular Formula	Nature of Molecules
1	6.902	1,2,4 Benzenetriol	126.00	6106391	C_6_H_6_O_3_	Phenol
2	6.902	3-Methyl-2-furoic acid	126.00	6106391	C_6_H_6_O_3_	Carboxylic acid
3	6.903	2,4-Octadienoic acid, 7-hydroxy-6-methyl	63.00	341570	C_9_H_14_O_3_	Unsaturated carboxylic acid
4	6.903	Diethyl mercaptal of d-mannose	63.00	341570	C_12_H_26_O_4_S_3_	Carbohydrate
5	8.469	1,2,4-Cyclopentanetriol	63.00	35297	C_5_H_10_O_3_	Cycloalkane
6	8.469	dl-Allo-cystathionine	63.00	35297	C_7_H_14_N_2_O_4_S	Amino acid
7	8.469	Phloroglucinol	63.00	35297	C_6_H_12_O_3_	Phenol
8	8.469	Acetoacetic acid, 1,3-dithio-, *S*-ethyl ester	63.00	35297	C_6_H_10_OS_2_	Ethyl ester of acetoacetic acid
9	8.469	1-Deoxy-d-arabitol	63.00	35297	C_5_H_12_O_4_	Sugar alcohol
10	8.383	β-d-Glucopyranose, 1,6-anhydro-	60.00	256706	C_6_H_10_O_5_	Carbohydrate
11	8.383	d-Mannoheptulose	60.00	256706	C_19_H_26_O_13_	Monosaccharide
12	8.383	D-Allose	60.00	256706	C_6_H_12_O_6_	Aldohexose sugar
13	8.383	d-erythro-Pentose, 2-deoxy-	60.00	256706	C_5_H_10_O_4_	Monosaccharide
14	8.628	Germacrene D	63.00	22058	C_15_H_24_	Sesquiterpene
15	8.628	Cis-muurola-3,5-diene	63.00	22058	C_15_H_24_	Isopropyl or carbocyclic compound
16	8.628	β-copaene	63.00	22058	C_15_H_24_	Sesquiterpene
17	9.872	Decanal	60.00	769010	C_10_H_20_O	Saturated fatty adehyde
18	9.872	Dodecanoic acid, 3-hydroxy-	60.00	769010	C_12_H_24_O_3_	Fatty acid
19	9.872	Butanoic acid, octyl ester	60.00	769010	C_12_H_24_O_2_	Carboxylic ester
20	9.872	Decanoic acid, 2-ethylhexyl ester	60.00	769010	C_18_H_36_O_2_	Carboxylic acid
21	9.872	Quinic acid	60.00	769010	C_7_H_12_O_6_	Carboxylic acid
22	9.872	1-Heptanol, 2,4-dimethyl-, (*R*,*R*)-(+)-	60.00	769010	C_9_H_20_O	Alcohol
23	9.872	d-Mannitol, 1-decylsulfonyl-	43.00	700880	C_16_H_34_O_7_S	Pentose alcohol
24	9.872	d-Mannitol, 1-thiohexyl-1-deoxy-	43.00	700880	C_12_H_26_O_5_S	Pentose alcohol
25	9.873	Sorbitol	73.00	166357	C_6_H_14_O_6_	Sugar alcohol
26	9.873	d-glycero-d-manno-Heptitol	73.00	166357	C_7_H_14_O_7_	Mannoheptulose
27	9.875	4-Diazodamantanone	44.00	117524	C_10_H_12_N_2_O	Ester
28	9.875	5alpha-Androstan-12-one, cyclic ethylene mercaptole	44.00	117524	C_21_H_34_S_2_	Terpenoid
29	11.013	3-Nonyn-2-ol	44.00	15411	C_9_H_16_O	Secondary alcohol
30	11.013	Pseduosarsasapogenin-5,20-dien	44.00	15411	C_27_H_42_O_3_	Sapogenins
31	11.013	Chlorozotocin	44.00	15411	C_9_H_16_ClN_3_O_7_	Amino sugar
32	11.013	Sparsomycin	44.00	15411	C_13_H_19_N_3_O_5_S_2_	Amino acid
33	11.860	l-Gala-l-ido-octose	73.00	77890	C_8_H_16_O_8_	Carbohydrate
34	12.284	9-Dodecen-1-ol, acetate, (*Z*)-	44.00	13463	C_14_H_26_O_2_	Diterpene
35	12.284	Cis-7-Tetradecen-1-ol	44.00	13463	C_14_H_28_O	Secondary alcohol
36	12.284	3-Chloropropionic acid, 10-undecenyl ester	44.00	13463	C_14_H_25_ClO_2_	Ester
37	12.284	Levomenthol	44.00	13463	C_10_H_20_O	Phenol
38	12.890	Dimethylmuconic acid	153.00	200436	C_8_H_10_O_4_	Ethyl ester
39	12.890	1,5-Hexadien-3-ol, trifluoroacetate	153.00	200436	C_8_H_9_F_3_O_2_	Ester
40	13.452	Hexadecanoic acid, methyl ester	74.00	620535	C_17_H_34_O_2_	Terpenoid
41	13.452	Tridecanoic acid, 12-methyl-, methyl ester	74.00	620535	C_17_H_34_O_2_	Terpenoid
42	13.452	Pentadecanoic acid, 14-methyl-, methyl ester	74.00	620535	C_17_H_34_O_2_	Terpenoid
43	13.452	Octadecanoic acid, 17-methyl-, methyl ester	74.00	620535	C_20_H_40_O_2_	Terpenoid
44	14.960	9,12-Octadecadienoic acid, methyl ester, (*E*,*E*)	44.00	41709	C_19_H_34_O_2_	Terpenoid
45	14.960	13-Tetradece-11-yn-1-ol	44.00	41709	C_14_H_24_O	Alcohol
46	14.960	11,14-Eicosadienoic acid, methyl ester	44.00	41709	C_21_H_38_O_2_	Terpenoid
47	14.960	9,12-Octadecadien-1-ol, (*Z*,*Z*)-	44.00	41709	C_18_H_34_O	Fatty alcohol
48	14.960	Linoelaidic acid	44.00	41709	C_18_H_32_O_2_	Fatty acid
49	14.960	9,12-Octadecadienoic acid (*Z*,*Z*)-	44.00	41709	C_18_H_32_O_2_	Fatty acid
50	15.233	8,11,14-Eicosatrienoic acid, (*Z*,*Z*,*Z*)-	55.00	118092	C_20_H_34_O_2_	Organic compound
51	15.336	Undecanal	71.00	97968	C_11_H_22_O	Organic compound
52	15.336	Dodecanal	71.00	97968	C_12_H_24_O	Aldehyde
53	15.156	1-Deoxy-d-arabitol	44.00	35550	C_5_H_12_O_4_	Secondary alcohol
54	20.205	Glycerol 1-palmitate	44.00	27326	C_19_H_38_O_4_	Saturated fatty acid
55	20.205	Octadecanoic acid, 2,3-dihydroxypropyl ester	44.00	27326	C_21_H_42_O_4_	Ester
56	24.520	13-Docosenamide, (*Z*)-	59.00	1596681	C_22_H_43_NO	Amines
57	24.520	Nonadecanamide	59.00	1596681	C_19_H_39_NO	Amines

R.T.: Retention time; *m*/*z*: *m* stands for mass and *z* stands for the charge number of ions.

**Table 2:** Effect of MECVL in the hole cross test.

Treatment	Dose	Number of Hole Crossed				
		0 min	30 min	60 min	90 min	120 min
Control	10 mL/kg	16.00 ± 0.32	12.40 ± 0.24	8.60 ± 0.24	6.20 ± 0.37	4.00 ± 0.32
Diazepam	1 mg/kg	13.40 ± 0.51 ***	5.60 ± 0.51 ***	4.00 ± 0.32 ***	2.00 ± 0.32 ***	0.78 ± 0.89 ***
MECVL	200	15.60 ± 0.51	11.40 ± 0.51	8.00 ± 0.32	4.60 ± 0.24 **	3.20 ± 0.37
MECVL	400	14.40 ± 0.24 *	7.40 ± 0.24 ***	6.20 ± 0.37 ***	3.80 ± 0.37 ***	2.40 ± 0.24 ***

Each value is presented as the mean ± SEM (n = 5). * *p* < 0.05, ** *p* < 0.01, and *** *p* < 0.001 compared with the control group (Dunnett’s test); MECVL: Methanolic extract of *Chukrasia velutina* leaves.

**Table 3:** Effect of MECVL in the open field test.

Treatment	Dose	Number of Squares Crossed				
		0 min	30 min	60 min	90 min	120 min
Control	10 mL/kg	75.60 ± 0.98	55.00 ± 0.71	53.00 ± 0.71	47.00 ± 0.71	37.00 ± 0.71
Diazepam	1 mg/kg	72.60 ± 0.93	51.00 ± 0.71 ***	27.00 ± 0.71 ***	17.00 ± 0.71 ***	13.00 ± 0.71 ***
MECVL	200	74.00 ± 0.71	54.00 ± 0.71	51.00 ± 1.58	42.00 ± 1.42	32.00 ± 0.95 ***
MECVL	400	73.00 ± 0.49	52.00 ± 0.86	49.00 ± 2.53	38.00 ± 2.53 ***	25.00 ± 0.86 ***

Each value is presented as the mean ± SEM (n = 5). *** *p* < 0.001 compared with the control group (Dunnett’s test); MECVL: Methanolic extract of *Chukrasia velutina* leaves.

**Table 4:** ADME/T properties of MECVL by SwissADME.

Compound Name	MW ^1^	HBA ^2^	HBD ^3^	LogP ^4^	MR ^5^	ROF ^6^
1,2,4 Benzenetriol (PubChem CID: 10787)	126.11	3	3	0.70	32.51	0
3-Methyl-2-furoic acid (PubChem CID: 78127)	126.11	3	1	0.97	30.63	0
2,4-Octadienoic acid, 7-hydroxy-6-methyl (PubChem CID: 5364229)	170.21	3	2	1.28	47.36	0
Diethyl mercaptal of d-mannose (PubChem CID: 124044)	330.53	4	4	1.44	87.22	0
dl-Allo-cystathionine (PubChem CID: 834)	222.26	6	4	−2.58	52.31	0
Phloroglucinol (PubChem CID: 230351)	132.16	3	3	−0.30	32.33	0
Acetoacetic acid, 1,3-dithio-, *S*-ethyl ester (PubChem CID: 547875)	162.27	1	0	1.92	46.54	0
β-d-Glucopyranose, 1,6-anhydro- (PubChem CID: 79029)	162.14	5	3	−1.26	32.38	0
d-Allose (PubChem CID: 439507)	180.16	6	5	−2.26	35.75	0
Germacrene D (PubChem CID: 5317570)	204.35	0	0	4.30	70.68	0
cis-muurola-3,5-diene (PubChem CID: 51351708)	204.35	0	0	4.14	69.04	0
β-copaene (PubChem CID: 87529)	204.35	0	0	4.40	67.14	0
Decanal (PubChem CID: 8175)	156.27	1	0	3.17	50.38	0
Dodecanoic acid, 3-hydroxy- (PubChem CID: 94216)	216.32	3	2	2.86	62.73	0
Butanoic acid, octyl ester (PubChem CID: 61030)	200.32	2	0	3.68	61.08	0
Quinic acid (PubChem CID: 6508)	192.17	6	5	−1.75	40.11	0
1-Heptanol, 2,4-dimethyl-, (*R*,*R*)-(+)- (PubChem CID: 87650)	144.25	1	1	2.62	46.54	0
d-Mannitol, 1-decylsulfonyl- (PubChem CID: 568528)	370.50	7	5	1.28	93.80	0
d-Mannitol, 1-thiohexyl-1-deoxy- (PubChem CID: 537501)	282.40	5	5	0.63	73.20	0
4-Diazodamantanone (PubChem CID: 561686)	176.22	3	0	1.28	47.69	0
3-Nonyn-2-ol (PubChem CID: 536232)	140.22	1	1	2.34	44.70	0
Chlorozotocin (PubChem CID: 451706)	313.69	8	5	−1.36	66.04	0
Sparsomycin (PubChem CID: 9543443)	361.44	5	4	0.01	91.61	0
9-Dodecen-1-ol, acetate, (Z)- (PubChem CID: 5363405)	226.36	2	0	4.11	70.22	0
Cis-7-Tetradecen-1-ol (PubChem ID: 5362795)	212.37	1	1	4.38	70.10	0
3-Chloropropionic acid, 10-undecenyl ester (PubChem ID: 543975)	260.80	2	0	4.58	75.02	0
Levomenthol (PubChem CID: 16666)	156.27	1	1	2.58	49.23	0
Dimethylmuconic acid (PubChem CID: 5369045)	170.16	4	2	0.83	43.17	0
1,5-Hexadien-3-ol, trifluoroacetate (PubChem CID: 238297)	194.15	5	0	2.21	40.69	0
Tridecanoic acid, 12-methyl-, methyl ester (PubChem CID: 21204)	242.40	2	0	4.75	75.50	0
13-Tetradece-11-yn-1-ol (PubChem CID: 543337)	208.34	1	1	4.12	68.26	0
Undecanal (PubChem CID: 8186)	170.29	1	0	3.55	55.19	0
Dodecanal (PubChem CID: 8194)	184.32	1	0	3.94	60.00	0
5-Butyl-1,3-oxathiolan-2-one (PubChem CID: 535042)	160.23	2	0	2.14	42.91	0
Glycerol 1-palmitate (PubChem CID: 14900)	330.50	4	2	4.64	97.06	0

^1^ MW: Molecular weight (acceptable range: <500 g/mol); ^2^ HBA: Hydrogen bond acceptor (acceptable range: ≤10); ^3^ HBD: Hydrogen bond donor (acceptable range: ≤5); ^4^ LogP: Lipophilicity (acceptable range: <5); ^5^ MR: Molar refractivity (range from 40 to 130); ^6^ ROF: Rule of five.

**Table 5:** Docking score of the selected compounds in MECVL against the human serotonin receptor (pdb: 5I6X), potassium channel receptor (pdb: 4UUJ), and human gabaa receptor (pdb: 4COF) for antidepressant, anxiolytic, and sedative activity, respectively.

Compounds Name	Docking Score (kcal/mol)		
	5I6X	4UUJ	4COF
1,2,4 Benzenetriol	**−5.18**	**−5.771**	**−5.447**
3-Methyl-2-furoic acid	−3.593	−4.085	**−5.608**
2,4-Octadienoic acid, 7-hydroxy-6-methyl	−2.416	−1.871	−3.355
Diethyl mercaptal of d-mannose	-	-	-
dl-Allo-cystathionine	-	-	-
Phloroglucinol	**−** **4.741**	**−5.955**	**−6.151**
Acetoacetic acid, 1,3-dithio-, S-ethyl ester	−3.145	−1.829	−4.042
β-d-Glucopyranose, 1,6-anhydro-	-	**−4.675**	**−5.895**
d-Allose	−3.192	−2.932	−5.357
Germacrene D	-	-	-
Cis-muurola-3,5-diene	-	-	-
β-copaene	-	-	-
Decanal	2.673	2.998	2.594
Dodecanoic acid, 3-hydroxy-	2.717	2.952	1.136
Butanoic acid, octyl ester	2.634	2.715	2.271
Quinic acid	-	**−4.42**	**−6.942**
1-Heptanol, 2,4-dimethyl-, (*R*,*R*)-(+)-	-	-	-
d-Mannitol, 1-decylsulfonyl-	-	-	-
d-Mannitol, 1-thiohexyl-1-deoxy-	-	-	0.428
4-Diazodamantanone	**−4.171**	−3.539	−3.734
3-Nonyn-2-ol	−1.407	−0.79	−0.909
Chlorozotocin	-	-	-
Sparsomycin	-	-	-
9-Dodecen-1-ol, acetate, (Z)-	-	-	-
Cis-7-Tetradecen-1-ol	-	-	-
3-Chloropropionic acid, 10-undecenyl ester	1.46	2.832	1.339
Levomenthol	**−3.911**	**−4.647**	−4.417
Dimethylmuconic acid	−2.71	−2.726	−4.814
1,5-Hexadien-3-ol, trifluoroacetate	-	-	-
Tridecanoic acid, 12-methyl-, methyl ester	1.42	2.602	1.292
13-Tetradece-11-yn-1-ol	4.354	4.228	4.67
Undecanal	2.431	3.537	2.216
Dodecanal	3.097	2.879	2.894
5-Butyl-1,3-oxathiolan-2-one	**−3.831**	−3.895	−4.03
Glycerol 1-palmitate	−0.992	0.902	−1.954
Reference drug (Imipramine/Diazepam)	−5.35	−4.035	−5.961

Bold text indicates the best docking scores.

**Table 6:** Biological activities of selected compounds from MECVL.

Compound Name	Biological Activity	References
1,2,4 Benzenetriol	Anti-microbial activity	[45]
3-Methyl-2-furoic acid	Anti-fungal and anti-tumor activity	[46]
2,4-Octadienoic acid, 7-hydroxy-6-methyl	Analgesic and anti-inflammatory activity	[47]
Diethyl mercaptal of d-mannose	Antibacterial and anti-fungal activity	[48]
dl-Allo-cystathionine	Analgesic, anti-inflammatory, antifungal, antibacterial activity	[49]
Phloroglucinol	Antioxidant, antineoplastic, anti-inflammatory, antimicrobial, antifungal activity	[50,51]
Acetoacetic acid, 1,3-dithio-, *S*-ethyl ester	Analgesic, antipyretic, antimalarial and antibiotic effect	[52,53]
β-D-Glucopyranose, 1,6-anhydro-	Anti-bacterial and antioxidant effect	[54,55]
D-Allose	Antioxidant, antibacterial and antiviral activity	[56,57]
Germacrene D	Neurological activity, cytotoxicity and antimicrobial activity	[58,59]
Cis-muurola-3,5-diene	Antimicrobial activity	[60]
β-copaene	Cytotoxic and antioxidant effect	[61,62]
Decanal	Antioxidant	[63]
Dodecanoic acid, 3-hydroxy-	Antibacterial, antitumor and antioxidant effect	[64]
Butanoic acid, octyl ester	Effect on CNS, antioxidant, analgesic and anti-inflammatory activity	[65,66]
Quinic acid	Antibacterial, antiviaral, antioxidant and hepatoprotective activity	[67,68,69]
1-Heptanol, 2,4-dimethyl-, (*R*,*R*)-(+)-	Antifungal activity	[70]
d-Mannitol, 1-decylsulfonyl-	Anti-diabetic, anti-microbial activity	[71,72]
d-Mannitol, 1-thiohexyl-1-deoxy-	Anti-microbial activity	[73]
4-Diazodamantanone	Antimicrobial, anti-inflammatory and antiacetylcholinesterase activities	[74,75]
3-Nonyn-2-ol	Anti-inflammatory, anti-septic, anti-tumor activity	[76,77]
Chlorozotocin	Anti-tumor, reduction in bone marrow toxicity	[78,79]
Sparsomycin	Anti-tumor antibiotic	[80]
9-Dodecen-1-ol, acetate, (*Z*)-	-	-
Cis-7-Tetradecen-1-ol	Anti-fungal, anti-bacterial, antioxidant, cytotoxic, anti-inflammatory, antinociceptive and hepatoprotective activty	[81,82,83,84]
3-Chloropropionic acid, 10-undecenyl ester	Antioxidant, anti-inflammatory, antitherogenic, hypocholesteromia activities, antibacterial activity	[85,86]
Levomenthol	Phytotoxic, antimicrobial, nematocidal, cytotoxic, anti-influenza and inflammation-promoting activities, antioxidant effect	[87,88]
Dimethylmuconic acid	-	-
1,5-Hexadien-3-ol, trifluoroacetate	Antioxidant Activity	[89]
Tridecanoic acid, 12-methyl-, methyl ester	Antimicrobial, antioxidant, and anti-inflammatory activities	[90]
13-Tetradece-11-yn-1-ol	Antibacterial, antifungal and antioxidant effect	[91]
Undecanal	Cytotoxic effect, antimicrobial, anti-inflammatory, and antioxidant activities	[92]
Dodecanal	Analgesic effect, antimicrobial, anti-inflammatory and cytotoxic activities	[93,94]
5-Butyl-1,3-oxathiolan-2-one	Anti-viral activity	[95]
Glycerol 1-palmitate	Palmitate-induced inflammatory effect on microphage	[96]

## Supplementary Materials

The following are available online. Table S1: Phytochemical screening of MECVL; Table S2: Binding interactions of the selected compounds against the human serotonin receptor (pdb: 5I6X) for antidepressant activity; Table S3: Binding interactions of the selected compounds against the potassium channel receptor (pdb: 4UUJ) for anxiolytic activity; Table S4: Binding interactions of the selected compounds against the human gabaa receptor (pdb: 4COF) for sedative activity; Figure S1: Best scored compounds exerted in 2D (a) and 3D (b) of 1,2,4-Benzenetriol (A), Phloroglucinol (B), 4-Diazodamantanone (C), Levomenthol (D), 5-Butyl-1,3-oxathiolan-2-one (E), and also (F) Imipramine (RSD) when they are enclosed with the human serotonin receptor (PDB: 5I6X) for antidepressant effect of MECVL; Figure S2: Best scored compounds exerted in 2D (a) and 3D (b) of Phloroglucinol (A), 1,2,4 Benzenetriol (B), β-d-Glucopyranose, 1,6-anhydro- (C), Levomenthol (D), Quinic acid (E), and also (F) Diazepam (RSD) when they are enclosed with the potassium channel receptor (PDB: 4UUJ) for anxiolytic effect of MECVL; Figure S3: Best scored compounds exerted in 2D (a) and 3D (b) of Quinic acid (A), Phloroglucinol (B), β-d-Glucopyranose, 1,6-anhydro- (C), 3-Methyl-2-furoic acid (D), 1,2,4 Benzenetriol (E), and also (F) Diazepam (RSD) when they were enclosed with the crystal structure of human gabaa receptor (PDB: 4COF) for sedative effect of MECVL.

## Abbreviations

MECVL: Methanolic extract of *Chukrasia velutina* leaves; GC-MS: Gas chromatography-mass spectrometer; CNS: Central nervous system; NEOA: Number of entries in the open; LSOA: Length of stay of the animal in the open arms; PDB: Protein data bank; ANOVA: Analysis of variance; SEM: Standard error mean; SPSS: Statistical package for Social Science; ADME/T: Absorption, Distribution, Metabolism, Excretion, and Toxicity.
