# Supplementary material for: GC-MS Phytochemical Profiling, Pharmacological Properties, and In Silico Studies of Chukrasia velutina Leaves: A Novel Source for Bioactive Agents
Source: Molecules. 2020 Aug 2;25(15):3536. doi: 10.3390/molecules25153536 (PMC7436235; doi:10.3390/molecules25153536)
Supplement: Supplementary file 1 [file molecules-25-03536-s001.pdf]

# Supplementary Materials

# GC-MS Phytochemical Profiling, Pharmacological Properties, and In Silico Studies of *Chukrasia velutina* Leaves: A Novel Source for Bioactive Agents

**Israt Jahan [1], Marzia Rahman Tona [1], Sanjida Sharmin [1,*], Mohammed Aktar Sayeed [1], Fatamatuz Zuhura Tania [1], Arkajyoti Paul [2,3], Md. Nazim Uddin Chy [1,2], Ahmed Rakib [4], Talha Bin Emran [2,3,*] and Jesus Simal-Gandara [5,*]**

[1] Department of Pharmacy, Faculty of Science and Engineering, International Islamic University Chittagong, Kumira, Chittagong 4318, Bangladesh; istiisrat@gmail.com (I.J.); marziaeva96@gmail.com (M.R.T.); sayeed_ustc@yahoo.com (M.A.S.); fatamaztaniaiiuc@gmail.com (F.Z.T.); nazim107282@gmail.com (M.N.U.C.)

[2] Drug Discovery, GUSTO A Research Group, Chittagong 4203, Bangladesh; arka.bgctub@gmail.com

[3] Department of Pharmacy, BGC Trust University Bangladesh, Chittagong 4381, Bangladesh

[4] Department of Pharmacy, Faculty of Biological Sciences, University of Chittagong, Chittagong 4331, Bangladesh; rakib.pharmacy.cu@gmail.com

[5] Nutrition and Bromatology Group, Department of Analytical and Food Chemistry, Faculty of 21 Food Science and Technology, University of Vigo–Ourense Campus, E32004 Ourense, Spain

* Correspondence: sharmin.shanjida@gmail.com (S.S.); talhabmb@bgctub.ac.bd (T.B.E.); jsimal@uvigo.es (J.S.-G.); Tel.: +88-01819-942214 (T.B.E.); +34-988-387000 (J.S.G.)

**Table S1.** Phytochemical screening of MECVL.

| Test Name | Observation |
|---|---|
| Resin | + |
| Flavonoids | - |
| Saponins | - |
| Phenol | ++ |
| Tannin | ++ |
| Alkaloid | + |
| Carbohydrate | ++ |
| Steroid | - |
| Cholesterol | - |
| Polyphenol | - |
| Glycoside | ++ |
| Lencoanthocyanin | - |

(+) = moderately present; (++) = highly present; (-) = absent.

**Table S2.** Binding interactions of the selected compounds against human serotonin receptor (pdb: 5I6X) for antidepressant activity.

| Compound Name | Hydrogen Bond Interactions | Hydrophobic Bond Interactions |
|---|---|---|
| 1,2,4 Benzenetriol | Gyl 476,<br>Gyl 476,<br>Ser 174 | Leu 492 (Pi-Sigma),<br>Val 479 (Pi- Alkyl) |
| 3-Methyl-2-furoic acid | Tyr 171 | Val 488 ( Alkyl),<br>Ile 581 (Alkyl),<br>Leu 492 (Alkyl),<br>Leu 492 (Pi- Alkyl),<br>Val 479 (Pi- Alkyl) |
| 2,4-Octadienoic acid, 7-hydroxy-6-methyl | Lue 248,<br>Lue 248,<br>Lue 245 | Lue 248 (Alkyl),<br>Gly 249 (Carbon) |
| Diethyl mercaptal of d-mannose | - | - |
| dl-Allo-cystathionine | - | - |
| Phloroglucinol | Ser 174,<br>Gly 476 | Val 429 (Pi- Alkyl),<br>Tyr 171 ( Pi-Pi-T Shaped) |
| Acetoacetic acid, 1,3-dithio-, S-ethyl ester | - | Ile 581 (Alkyl) |
| .beta.-D-Glucopyranose, 1,6-anhydro- | - | - |
| D-Allose | Try 171 | - |
| Germacrene D | - | - |
| Cis-muurola-3,5-diene | - | - |
| .beta.-copaene | - | - |
| Decanal | Gln 246<br>Gln 246 | Trp 573 (Pi- Alkyl),<br>Trp 573 (Pi- Alkyl) |
| Dodecanoic acid, 3-hydroxy- | - | Val 479 (Alkyl),<br>Val 488 (Alkyl) |
| Butanoic acid, octyl ester | - | Trp 573 (Pi- Alkyl),<br>Trp 573 (Pi- Alkyl) |
| Quinic acid | - | - |
| 1-Heptanol, 2,4-dimethyl-, (R,R)-(+)- | - | - |
| d-Mannitol, 1-decylsulfonyl- | - | - |
| d-Mannitol, 1-thiohexyl-1-deoxy- | - | - |
| 4-Diazodamantanone | Tyr 171 | Val 479 (Alkyl),<br>Leu 492 (Alkyl) |
| 3-Nonyn-2-ol | Gln 246 | Gln 246 (Carbon)<br>Leu 245 (Carbon)<br>Leu 244 (Alkyl)<br>Val 479 (Alkyl)<br>Val 488 (Alkyl) |
| Chlorozotocin | - | - |
| Sparsomycin | - | - |
| 9-Dodecen-1-ol, acetate, (Z)- | - | - |
| Cis-7-Tetradecen-1-ol | - | - |

| | | |
|---|---|---|
| 3-Chloropropionic acid, 10-undecenyl ester | - | Leu 245 (Alkyl)<br>Leu 491 (Alkyl)<br>Leu 577 (Alkyl)<br>Val 488 (Alkyl) |
| Levomenthol | - | Val 479 (Alkyl)<br>Val 488 (Alkyl)<br>Val 488 (Alkyl)<br>Leu 245 (Alkyl)<br>Leu 577 (Alkyl)<br>Leu 577 (Alkyl)<br>Trp 573 (Pi- Alkyl)<br>Trp 573 (Pi- Alkyl) |
| Dimethylmuconic acid | Tyr 171 | Leu 577 (Alkyl)<br>Ile 581 (Alkyl)<br>Phe 170 (Pi-Alkyl) |
| 1,5-Hexadien-3-ol, trifluoroacetate | - | - |
| Tridecanoic acid, 12-methyl-, methyl ester | - | Leu 248 (Alkyl)<br>Val 479 (Alkyl)<br>Gly 476 (Carbon)<br>Gly 476 (Carbon) |
| 13-Tetradece-11-yn-1-ol | - | - |
| Undecanal | Gln 246<br>Trp 573 | Trp 573 (Pi-Alkyl)<br>Trp 573 (Pi-Alkyl)<br>Trp 573 (Pi-Sigma) |
| Dodecanal | Gln 246<br>Trp 573 | Ile 576 (Alkyl)<br>Trp 573 (Pi-Alkyl)<br>Trp 573 (Pi-Alkyl) |
| 5-Butyl-1,3-oxathiolan-2-one | Tyr 171 | Leu 245 (Alkyl)<br>Leu 248 (Alkyl)<br>Val 479 (Alkyl)<br>Val 488 (Alkyl)<br>Tyr 171 (Pi-Sulfur) |
| Glycerol 1-palmitate | - | Leu 245 (Alkyl)<br>Leu 491 (Alkyl)<br>Leu 577 (Alkyl)<br>Val 488 (Alkyl)<br>Ala 580 (Carbon) |
| Reference drug (Imipramine) | Tyr 171 | Val 479 (Pi-Alkyl)<br>Val 479 (Pi-Alkyl)<br>Val 479 (Alkyl)<br>Leu 492 (Alkyl)<br>Ile 581 (Alkyl) |

**Table S3.** Binding interactions of the selected compounds against potassium channel receptor (pdb: 4UUJ) for anxiolytic activity.

| Compound Name | Hydrogen Bond Interactions | Hydrophobic Bond Interactions |
|---|---|---|
| 1,2,4 Benzenetriol | Thr 72<br>Gly 79<br>Leu 81 | Gly 79 (Carbon) |
| 3-Methyl-2-furoic acid | Trp 68<br>Leu 81 | Met 96 (Alkyl)<br>Gly 77 (Carbon)<br>Tyr 82 (Carbon) |
| 2,4-Octadienoic acid, 7-hydroxy-6-methyl | - | Tyr 82 (Carbon)<br>Trp 68 (Pi-Alkyl) |
| Diethyl mercaptal of d-mannose | - | - |
| dl-Allo-cystathionine | - | - |
| Phloroglucinol | Glu 71<br>Thr 72<br>Gly 79 | - |
| Acetoacetic acid, 1,3-dithio-, S-ethyl ester | - | Trp 68 (Pi-Alkyl) |
| .beta.-D-Glucopyranose, 1,6-anhydro- | Trp 68<br>Thr 72<br>Leu 81 | Gly 77 (Carbon)<br>Gly 79 (Carbon) |
| D-Allose | Thr 75 | Gly 77 (Carbon)<br>Gly 77 (Carbon) |
| Germacrene D | - | - |
| Cis-muurola-3,5-diene | - | - |
| .beta.-copaene | - | - |
| Decanal | - | Pro 83 (Alkyl)<br>Met 96 (Alkyl)<br>Ala 92 (Alkyl)<br>Trp 68 (Pi-Alkyl) |
| Dodecanoic acid, 3-hydroxy- | Trp 68 | Trp 68 (Pi-Alkyl)<br>Pro 83 (Carbon)<br>Tyr 82 (Pi-Alkyl) |
| Butanoic acid, octyl ester | - | Ala 92 (Alkyl)<br>Pro 82 (Alkyl) |
| Quinic acid | Trp 68<br>Gly 77 | Gly 77 (Carbon)<br>Gly 77 (Carbon)<br>Pro 83 (Carbon)<br>Tyr 82 (Carbon) |
| 1-Heptanol, 2,4-dimethyl-, (R,R)-(+)- | - | - |
| d-Mannitol, 1-decylsulfonyl- | - | - |
| d-Mannitol, 1-thiohexyl-1-deoxy- | - | - |
| 4-Diazodamantanone | - | Leu 81 (Pi-Alkyl) |
| 3-Nonyn-2-ol | - | Met 96 (Alkyl) |
| Chlorozotocin | - | - |

| | | |
|---|---|---|
| Sparsomycin | - | - |
| 9-Dodecen-1-ol, acetate, (Z)- | - | - |
| cis-7-Tetradecen-1-ol | - | - |
| 3-Chloropropionic acid, 10-undecenyl ester | - | Tyr 82 (Pi- Sigma) |
| Levomenthol | Leu 81 | Met 96 (Alkyl)<br>Met 96 (Alkyl)<br>Ala 92 (Alkyl)<br>Pro 83 (Alkyl)<br>Trp 68 (Pi-Alkyl)<br>Trp 68 (Pi-Alkyl)<br>Tyr 82 (Pi-Alkyl)<br>Tyr 82 (Pi-Alkyl)<br>Gly 77 (Carbon) |
| Dimethylmuconic acid | Thr 72<br>Trp 68 | Gly 77 (Carbon)<br>Gly 77 (Carbon) |
| 1,5-Hexadien-3-ol, trifluoroacetate | - | - |
| Tridecanoic acid, 12-methyl-, methyl ester | - | Trp 68 (Pi-Alkyl)<br>Trp 68 (Pi-Alkyl)<br>Ala 92 (Pi-Alkyl)<br>Met 96 (Alkyl)<br>Pro 83 (Alkyl) |
| 13-Tetradece-11-yn-1-ol | - | Ala 92 (Alkyl)<br>Pro 83 (Alkyl)<br>Arg 89 (Alkyl)<br>Gly 77 (Carbon) |
| Undecanal | - | Trp 68 (Pi-Alkyl)<br>Trp 68 (Pi-Alkyl)<br>Pro 83 (Alkyl)<br>Ala 92 (Alkyl)<br>Met 96 (Alkyl) |
| Dodecanal | - | Gly 79 (Carbon)<br>Arg 89 (Alkyl) |
| 5-Butyl-1,3-oxathiolan-2-one | Trp 68 | Pro 83 (Carbon)<br>Pro 83 (Carbon)<br>Trp 68 (Pi-Alkyl) |
| Glycerol 1-palmitate | Trp 78 | Trp 78 (Pi-Alkyl)<br>Trp 78 (Pi-Sigma)<br>Gly 79 (Carbon) |
| Reference drug (Diazepam) | - | Trp 163 (Pi-Pi-Stacked)<br>Trp 163 (Pi-Pi-Stacked)<br>Trp 163 (Pi-Pi-T Shaped)<br>Trp 163 (Pi-Pi-T Shaped)<br>Asp 165 (Carbon)<br>Thr 164 (Carbon)<br>Lys 142 (Pi-Alkyl)<br>Asp 143 (Pi-Anion) |

**Table S4.** Binding interactions of the selected compounds against human gabaa receptor (pdb: 4COF) for sedative activity.

| Compound Name | Hydrogen Bond Interactions | Hydrophobic Bond Interactions |
|---|---|---|
| 1,2,4 Benzenetriol | Glu 155 | Tyr 62 (Pi-Pi- stacked) |
| | Glu 155 | Tyr 205 (Pi-Pi-T- Shaped) |
| | Gln 64 | |
| 3-Methyl-2-furoic acid | Trp 68 | Met 96 (Alkyl) |
| | Leu 81 | Gly 77 (Carbon) |
| | | Tyr 82 (Carbon) |
| 2,4-Octadienoic acid, 7-hydroxy-6-methyl | Gln 64 | Asp 43 (Carbon) |
| | | Tyr 157 (Carbon) |
| | | Tyr 157 (Pi-Alkyl) |
| | | Tyr 97 (Pi-Alkyl) |
| | | Tyr 62 (Pi-Alkyl) |
| | | Phr 200 (Pi-Alkyl) |
| Diethyl mercaptal of d-mannose | - | - |
| dl-Allo-cystathionine | - | - |
| Phloroglucinol | Gln 64 | Tyr 157 (Pi-Pi-T- Shaped) |
| | Thr 202 | Phe 200 (Pi-Pi-T- Shaped) |
| | Glu 155 | |
| | Tyr 97 | |
| Acetoacetic acid, 1,3-dithio-, S-ethyl ester | Gln 64 | Tyr 97 (Pi-Alkyl) |
| | Gln 64 | Tyr 205 (Pi-Alkyl) |
| .beta.-D-Glucopyranose, 1,6-anhydro- | Gln 64 | Glu 155 (Carbon) |
| | Thr 202 | Glu 155 (Carbon) |
| | Glu 155 | Tyr 157 (Carbon) |
| | Tyr 97 | |
| | Tyr 157 | |
| D-Allose | Glu 155 | Tyr 157 (Carbon) |
| Germacrene D | - | - |
| Cis-muurola-3,5-diene | - | - |
| .beta.-copaene | - | - |
| Decanal | Gln 64 | Phe 200 (Pi-Alkyl) |
| Dodecanoic acid, 3-hydroxy- | Tyr 157 | Tyr 62 (Pi-Alkyl) |
| | Tyr 157 | Leu 99 (Alkyl) |
| | Gln 64 | Tyr 157 (Carbon) |
| Butanoic acid, octyl ester | Gln 64 | Ala 45 (Alkyl) |
| | | Leu 99 (Alkyl) |
| | | Met 115 (Alkyl) |
| | | Tyr 205 (Pi-Alkyl) |
| Quinic acid | Gln 64 | Tyr 157 (Carbon) |
| | Tyr 157 | |
| | Tyr 205 | |
| | Thr 202 | |
| | Glu 155 | |

| | | |
|---|---|---|
| | Glu 155 | |
| 1-Heptanol, 2,4-dimethyl-, (R,R)-(+)- | - | - |
| d-Mannitol, 1-decylsulfonyl- | - | - |
| d-Mannitol, 1-thiohexyl-1-deoxy- | Gln 64 | Tyr 62 (Pi-Sulfur) |
| | Tyr 205 | Phe 200 (Pi-Sulfur) |
| | | Leu 99 (Pi-Alkyl) |
| | | Thr 202 (Carbon) |
| | | Thr 202 (Carbon) |
| | | Tyr 157 (Carbon) |
| | | Tyr 205 (Carbon) |
| 4-Diazodamantanone | - | Tyr 155 (Pi-Cation) |
| | | Tyr 62 (Pi-Alkyl) |
| | | Tyr 205 (Pi-Alkyl) |
| | | Phe 200 (Pi-Alkyl) |
| 3-Nonyn-2-ol | Glu 155 | Met 155 (Pi-Alkyl) |
| | | Tyr 205 (Pi-Alkyl) |
| | | Glu 155 (Carbon) |
| Chlorozotocin | - | - |
| Sparsomycin | - | - |
| 9-Dodecen-1-ol, acetate, (Z)- | - | - |
| Cis-7-Tetradecen-1-ol | - | - |
| 3-Chloropropionic acid, 10-undecenyl ester | Gln 64 | Met 115 (Cl, Br, I)) |
| Levomenthol | Gln 64 | Tyr 62 (Pi-Alkyl) |
| | | Tyr 97 (Pi-Alkyl) |
| | | Tyr 205 (Pi-Alkyl) |
| | | Tyr 205 (Pi-Alkyl) |
| | | Phe 200 (Pi-Alkyl) |
| Dimethylmuconic acid | Gln 64, Glu 155, Tyr 157 (2) | Tyr 92 (Pi-Alkyl) |
| | | Tyr 157 (Pi-Alkyl) |
| | | Phe 200 (Pi-Alkyl) |
| | | Gly 127 (Pi-Alkyl) |
| 1,5-Hexadien-3-ol, trifluoroacetate | - | - |
| Tridecanoic acid, 12-methyl-, methyl ester | Ala 201 | Tyr 97 (Pi-Alkyl) |
| | | Tyr 205 (Pi-Alkyl) |
| | | Tyr 205 (Pi-Alkyl) |
| | | Phe 200 (Pi-Alkyl) |
| | | Phe 200 (Pi-Sigma) |
| | | Ala 201 (Alkyl) |
| 13-Tetradece-11-yn-1-ol | - | Tyr 62 (Pi-Sigma) |
| | | Tyr 157 (Pi-Alkyl) |
| | | Met 115 (Alkyl) |
| Undecanal | Gln 64 | Ala 45 (Pi-Alkyl) |
| | | Tyr 62 (Pi-Alkyl) |
| Dodecanal | Tyr 157 | Tyr 62 (Pi-Alkyl) |
| | | Gly 127 (Carbon) |
| 5-Butyl-1,3-oxathiolan-2-one | Gln 64 | Tyr 62 (Pi-Alkyl) |

| | | |
|---|---|---|
| | | Tyr 205 (Pi-Alkyl) |
| | | Phe 200 (Pi-Alkyl) |
| Glycerol 1-palmitate | Ala 45 | Asp 43 (Carbon) |
| | Ile 44 | Asp 43 (Acceptor-Acceptor) |
| | Arg 207 | Phe 200 (Pi-Sigma) |
| Reference drug (Diazepam) | - | Tyr 157 (Carbon) |
| | | Tyr 157 (Carbon) |
| | | Tyr 157 (Carbon) |
| | | Tyr 205 (Pi-Alkyl) |
| | | Ala 201 (Pi-Alkyl) |
| | | Asp 43 (Pi-Anion) |
| | | Tyr 62 (Pi-Pi-Stacked) |
| | | Phe 200 (Pi-Pi-Stacked) |

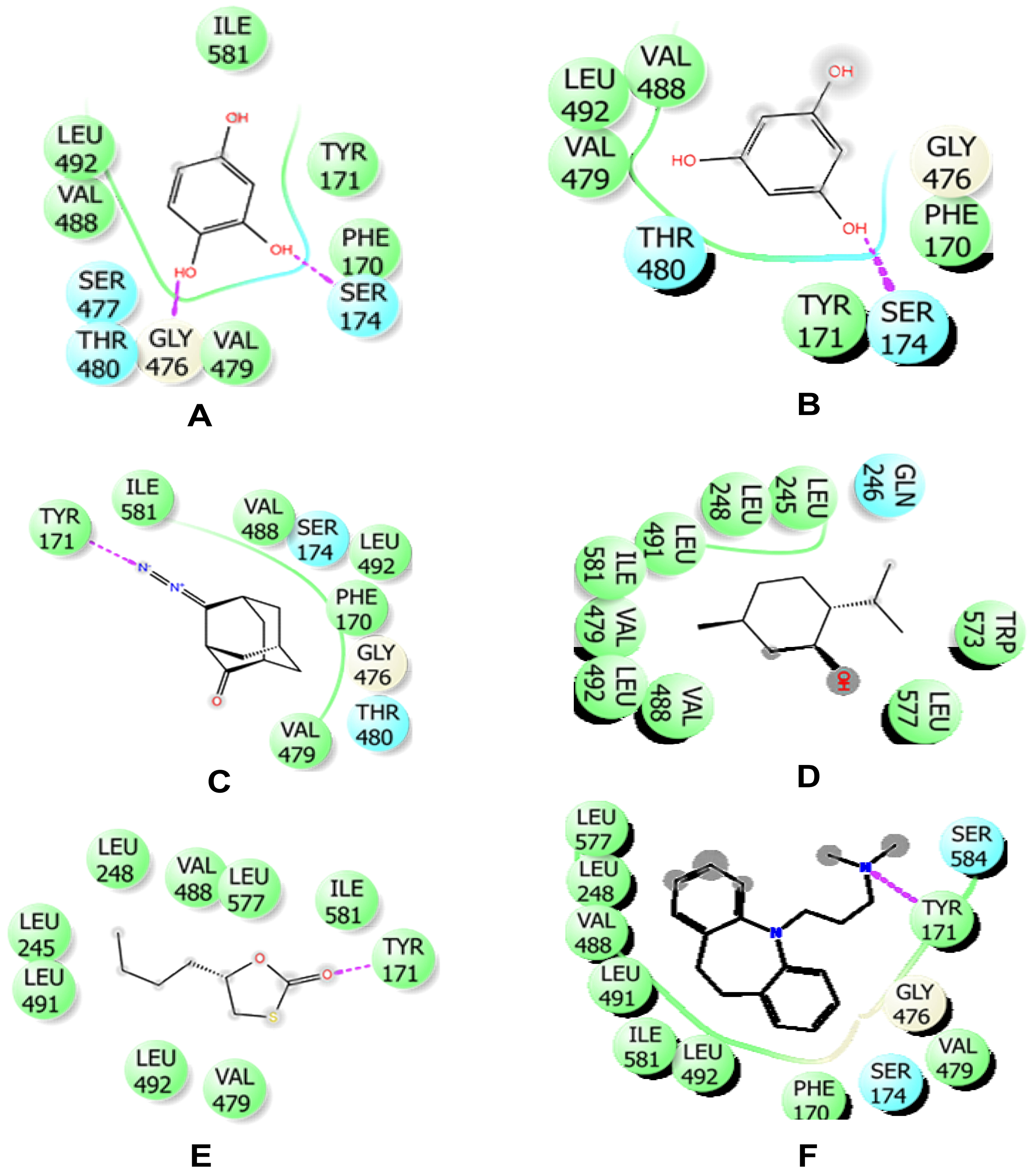
A
ILE 581
LEU 492
VAL 488
TYR 171
PHE 170
SER 477
SER 174
THR 480
GLY 476
VAL 479
OH
HO
B
LEU 492
VAL 488
VAL 479
GLY 476
PHE 170
THR 480
TYR 171
SER 174
OH
HO
C
TYR 171
ILE 581
VAL 488
SER 174
LEU 492
PHE 170
GLY 476
VAL 479
THR 480
D
LEU 245
LEU 248
GLN 246
LEU 491
ILE 581
VAL 479
LEU 492
VAL 488
TRP 573
LEU 577
E
LEU 248
VAL 488
LEU 577
ILE 581
LEU 245
LEU 491
TYR 171
LEU 492
VAL 479
F
LEU 577
LEU 248
VAL 488
LEU 491
ILE 581
LEU 492
PHE 170
SER 174
SER 584
TYR 171
GLY 476
VAL 479

**(a)**

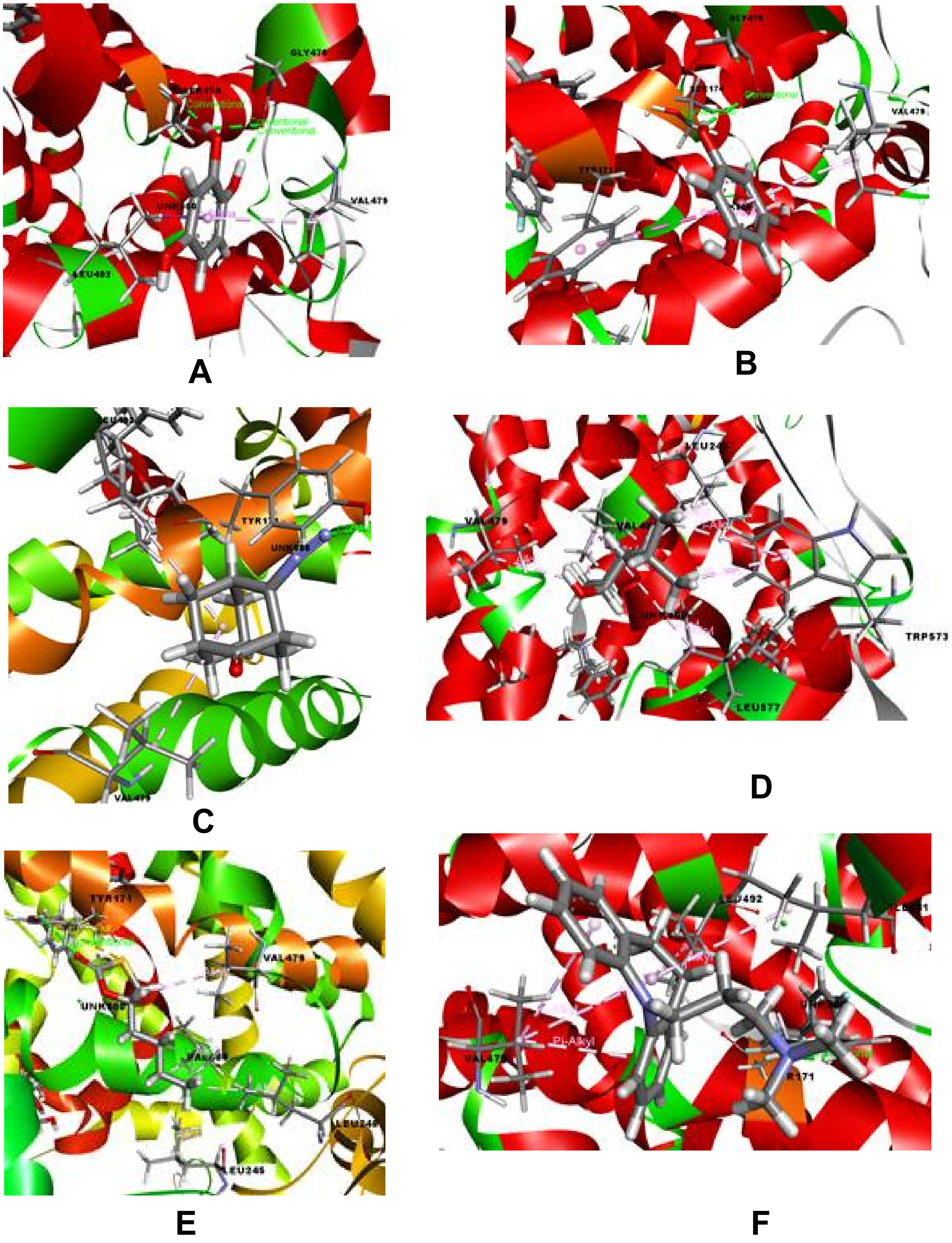


(b)

**Figure S1.** Best scored compounds exert in 2D **(a)** and 3D **(b)** of 1,2,4-Benzenetriol **(A),** Phloroglucinol **(B),** 4-Diazodamantanone **(C),** Levomenthol **(D),** 5-Butyl-1,3-oxathiolan-2-one **(E)** and also **(F)** Imipramine (RSD) when they enclosed with human serotonin receptor (PDB: 5I6X) for antidepressant effect of MECVL.

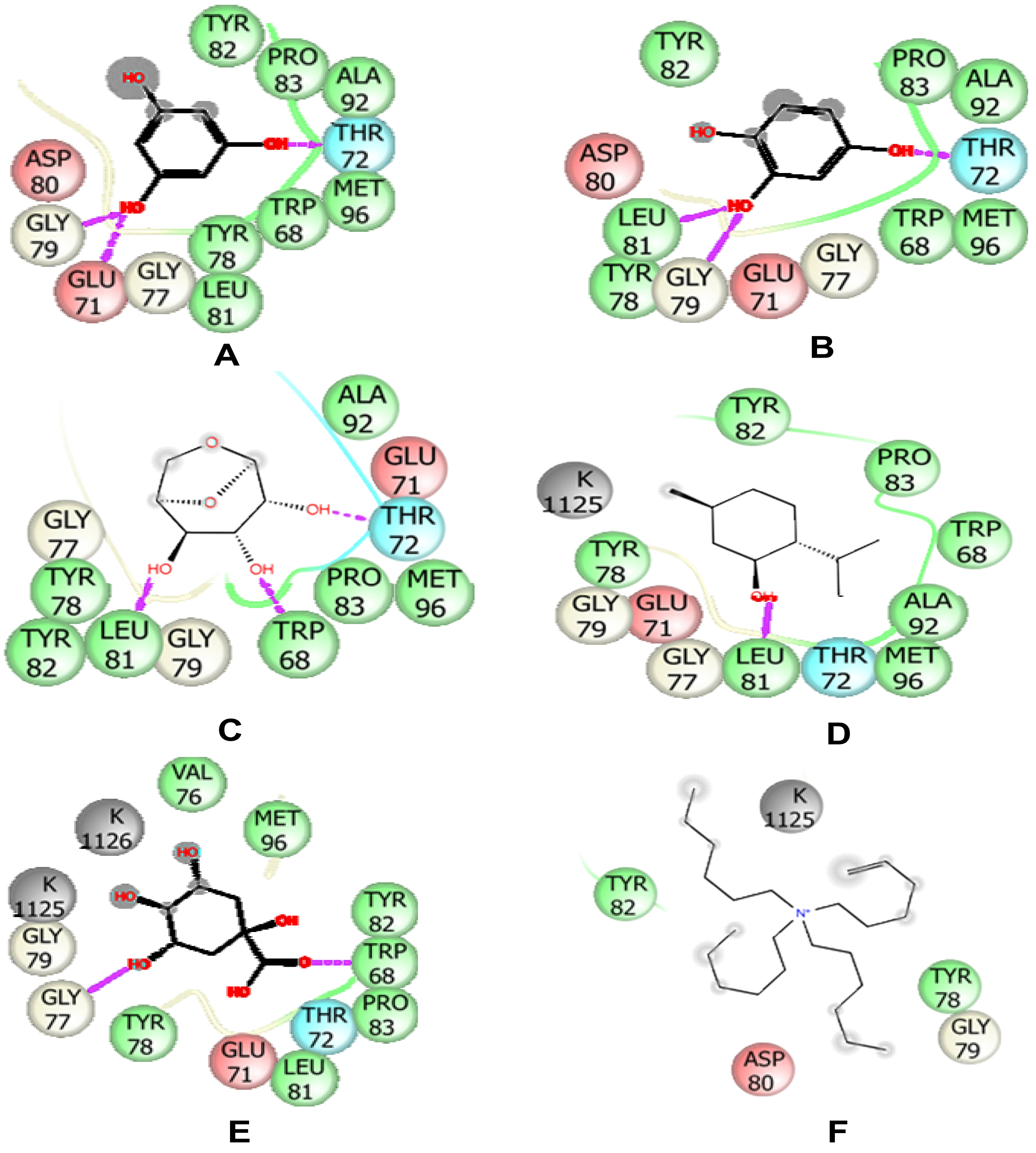
A
TYR 82
PRO 83
ALA 92
THR 72
MET 96
TRP 68
TYR 78
LEU 81
GLY 77
GLU 71
GLY 79
ASP 80
B
TYR 82
PRO 83
ALA 92
THR 72
ASP 80
LEU 81
TRP 68
MET 96
TYR 78
GLY 79
GLU 71
GLY 77
C
ALA 92
GLU 71
THR 72
GLY 77
TYR 78
PRO 83
MET 96
TYR 82
LEU 81
GLY 79
TRP 68
D
TYR 82
PRO 83
K 1125
TRP 68
TYR 78
ALA 92
GLY 79
GLU 71
GLY 77
LEU 81
THR 72
MET 96
E
VAL 76
K 1126
MET 96
K 1125
GLY 79
TYR 82
TRP 68
GLY 77
PRO 83
THR 72
TYR 78
GLU 71
LEU 81
F
K 1125
TYR 82
TYR 78
GLY 79
ASP 80

**(a)**

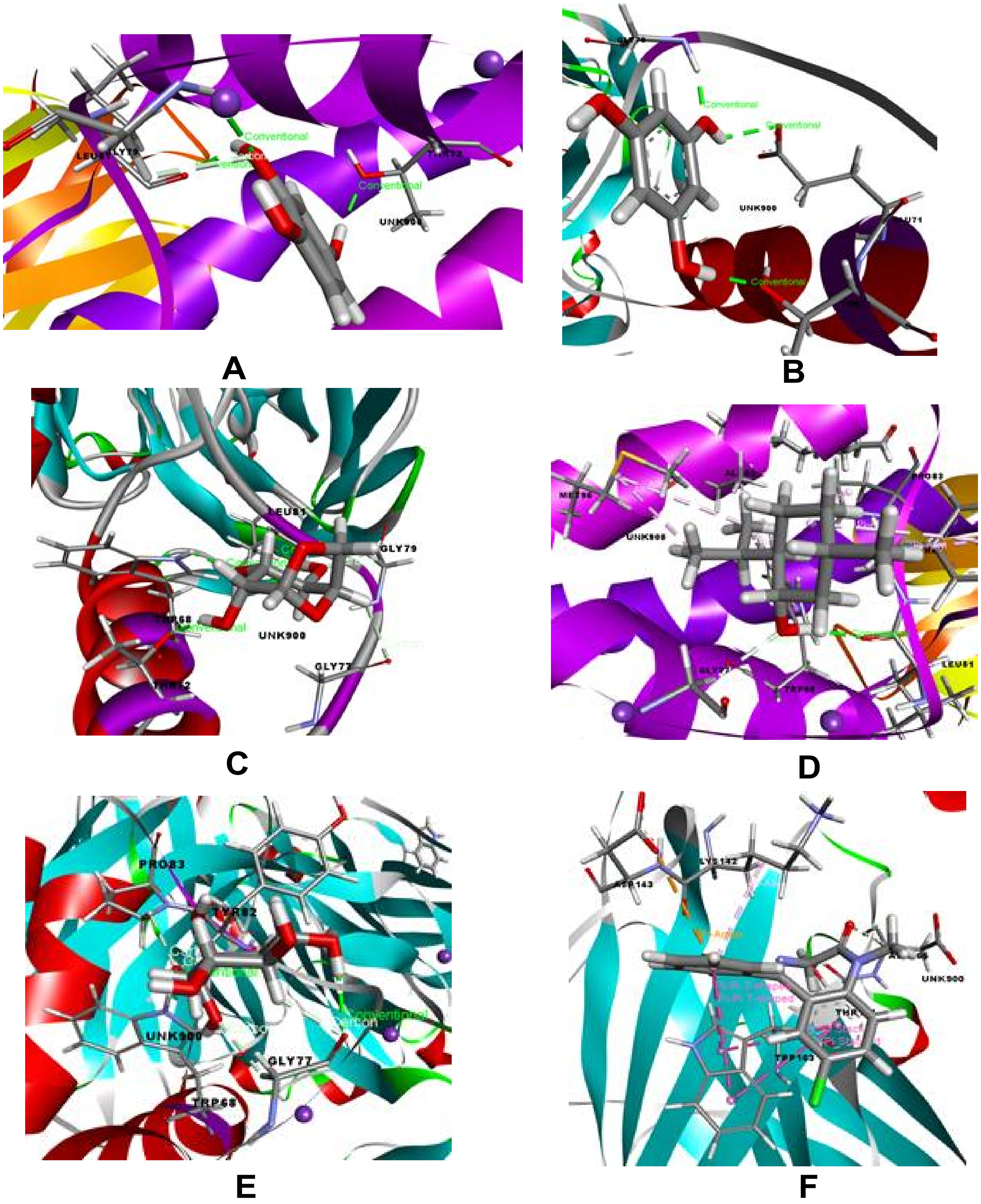


**(b)**

**Figure S2.** Best scored compounds exert in 2D **(a)** and 3D **(b)** of Phloroglucinol (**A**)**,** 1,2,4 Benzenetriol (**B**)**,** .beta.-D-Glucopyranose, 1,6-anhydro- (**C**)**,** Levomenthol **(D),** Quinic acid **(E)** and also **(F)** Diazepam (RSD) when they enclosed with potassium channel receptor (PDB: 4UUJ) for anxiolytic effect of MECVL.

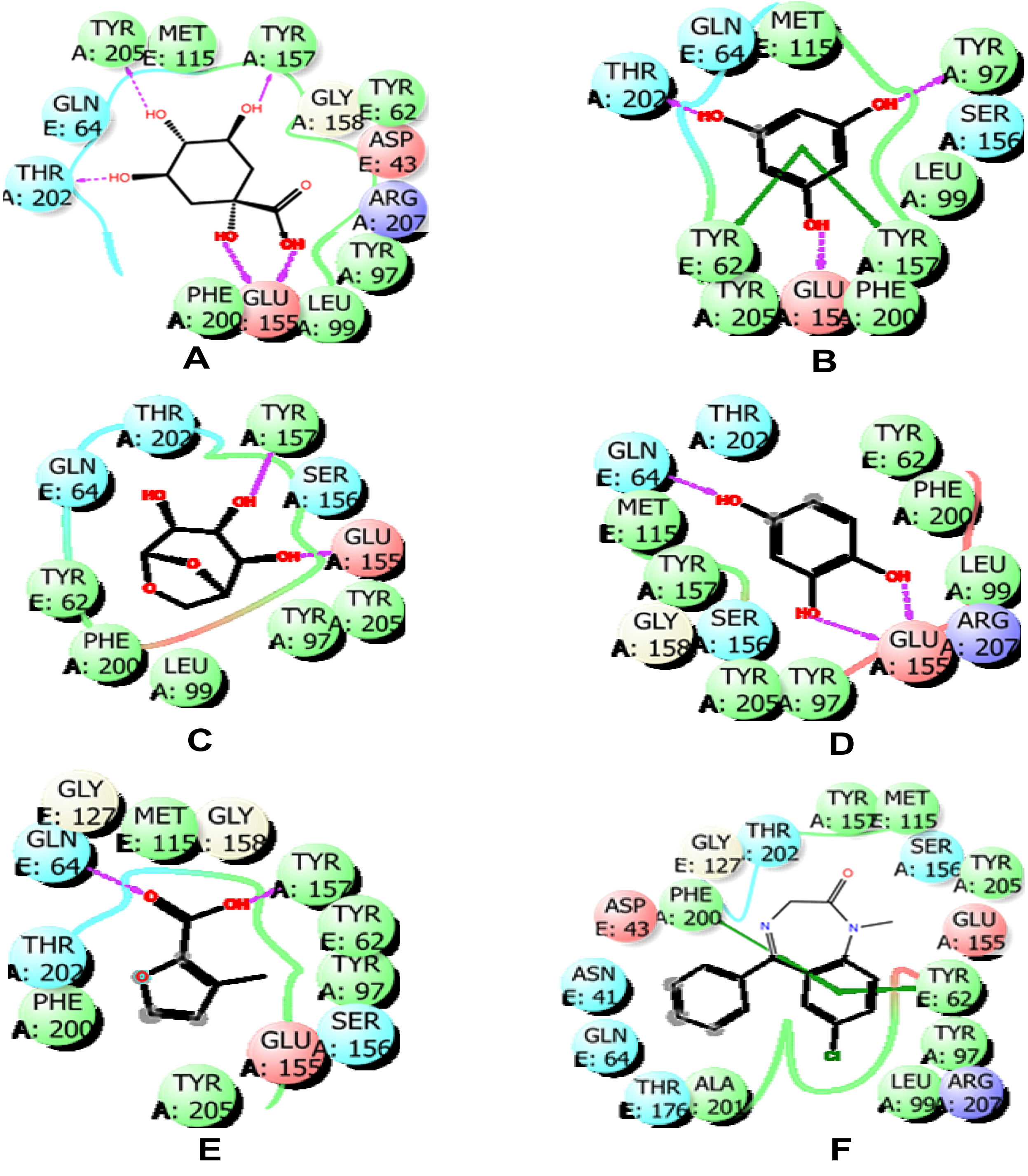
A
B
C
D
E
F
TYR
MET
GLN
THR
GLY
ASP
ARG
PHE
GLU
LEU
SER
ASN
ALA
HO
OH
O
N
Cl

**(a)**

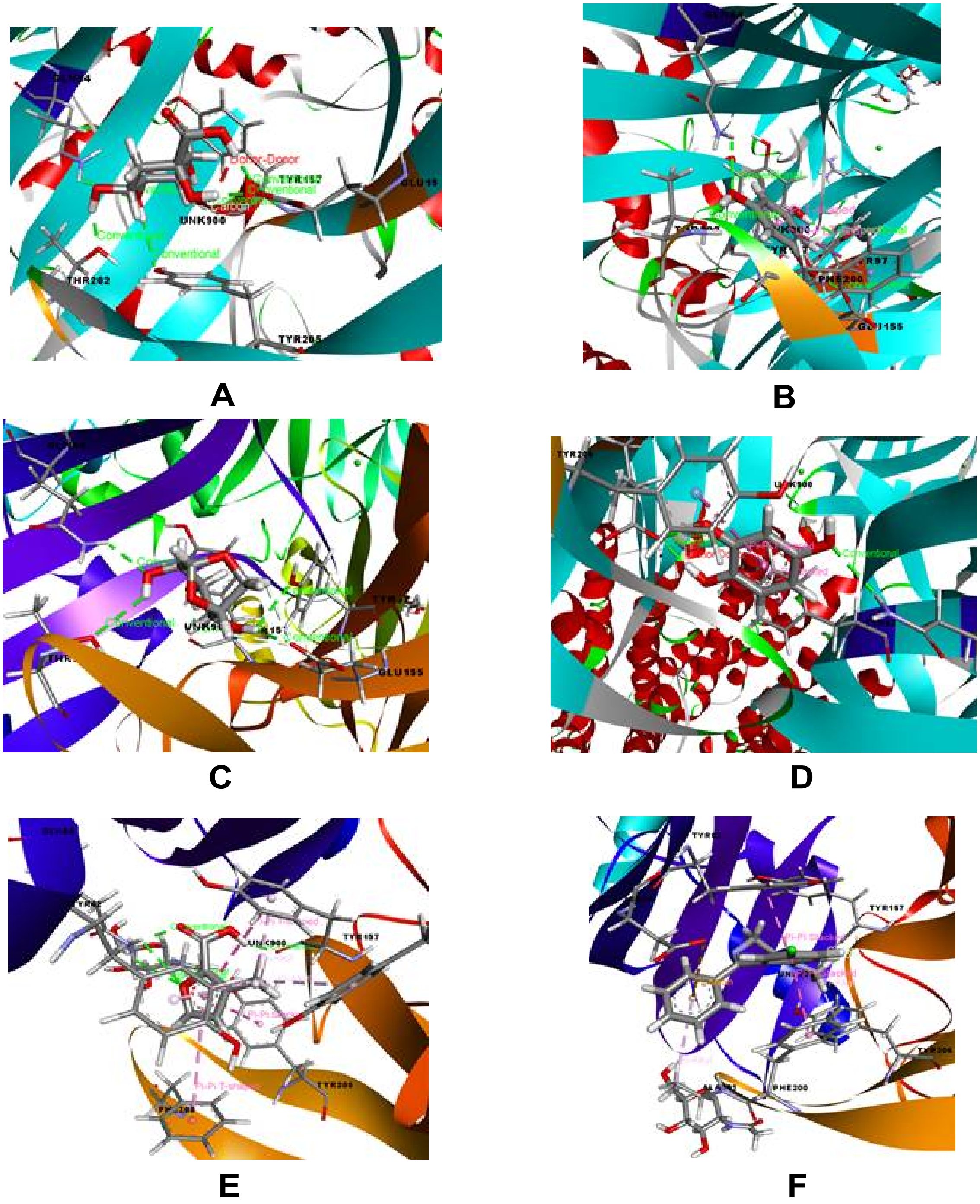


**(b)**

**Figure S3.** Best scored compounds exert in 2D **(a)** and 3D **(b)** of Quinic acid **(A),** Phloroglucinol **(B),** .beta.-D-Glucopyranose, 1,6-anhydro- **(C),** 3-Methyl-2-furoic acid **(D),** 1,2,4 Benzenetriol **(E)** and also **(F)** Diazepam (RSD) when they enclosed with crystal structure of human gabaa receptor (PDB: 4COF) for sedative effect of MECVL.
